# A photoswitchable strapped calix[4]pyrrole receptor: highly effective chloride binding and release†

**DOI:** 10.1039/d0sc06686a

**Published:** 2021-01-13

**Authors:** David Villarón, Maxime A. Siegler, Sander J. Wezenberg

**Affiliations:** Leiden Institute of Chemistry, Leiden University Einsteinweg 55 2333 CC Leiden The Netherlands s.j.wezenberg@lic.leidenuniv.nl; Department of Chemistry, Johns Hopkins University 3400 N. Charles St. Baltimore MD 21218 USA

## Abstract

A stiff-stilbene strapped calix[4]pyrrole receptor can be reversibly switched by light between a strong chloride-binding *Z*-isomer and a very weakly binding *E*-isomer. The light-induced switching process is monitored by UV-Vis and ^1^H NMR spectroscopy and chloride binding is studied in detail using both ^1^H NMR and ITC titrations in DMSO and MeCN. In DMSO, at millimolar concentrations, switching from a fully bound to an almost fully unbound state can be triggered. Quantification of the binding constants in MeCN reveals an extraordinary 8000-fold affinity difference between the *Z*- and *E*-isomer. Single crystal X-ray crystallographic analysis gives insight into the structure of the photogenerated *E*-isomer and the geometry of the chloride-bound receptors is optimized by DFT calculations. The highly effective control of binding affinity demonstrated in this work opens up new prospects for on demand binding and release in extractions and photocontrol of membrane transport processes, among other applications.

## Abstract 2

A reconfigurable calix[4]pyrrole receptor containing a stiff-stilbene strap can be switched by light between a strong and weak binding form, showing an 8000-fold affinity difference for chloride.

## Graphical abstract

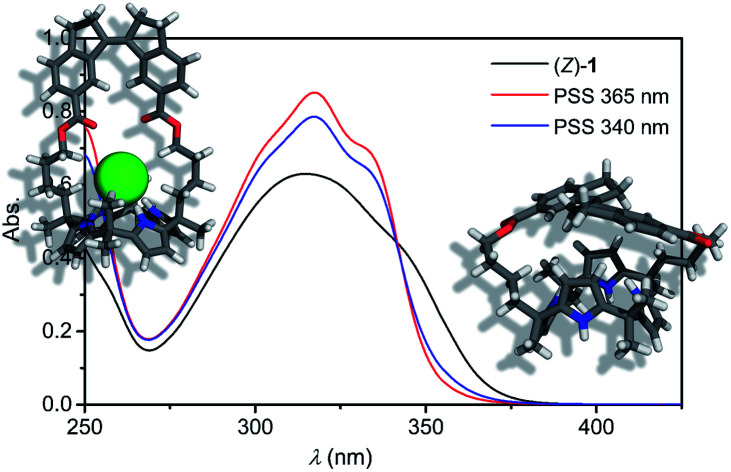

## Introduction

Within the field of supramolecular chemistry, the design and synthesis of anion receptors is one of the most vibrant areas of research.^1^ Synthetic anion receptors have been successfully applied in, for instance, analyte sensing,^2^ waste extractions,^3^ and transmembrane transport.^4,5^ In such applications, control over binding properties by external stimuli will provide many advantages. For example, separation/recovery of the receptor and the substrate in extractions would be facilitated and membrane transport processes could be activated/deactivated at will. Not surprisingly, therefore, several strategies to modulate anion-binding properties have been developed over the past decade, most notably based on a light stimulus.^6,7^ Nevertheless, to achieve a sizeable difference in binding affinity between the photoaddressable states remains highly difficult. Furthermore, quantitative switching to an inactive (*i.e.*, weak or non-binding) form would be essential for full recovery in extractions and deactivation of transport. However, in currently known systems, the low-affinity form is either still significantly capable of binding or a considerable amount of the high-affinity form remains present at photoequilibrium.

Among the most widely studied anion receptors in literature is calix[4]pyrrole, which is a macrocycle that strongly interacts with a number of small anions, most notably chloride.^8^ A very effective strategy to enhance their guest-binding affinity, as was first described by the groups of Lee and Sessler, is to bridge the distal *meso*-positions, providing an isolated binding domain. These bridged systems have been termed “strapped calix[4]pyrroles” and they are receiving rapidly increasing attention.^9,10^ As their binding properties were found to largely depend on the nature and the length of the strap, we became interested in incorporating a molecular photoswitch to allow structure post-modification. As far as we know, strap modulation by light has not yet been explored in these receptors. Our photoswitch of choice was stiff-stilbene because of its high thermal stability and large geometrical change upon isomerization.^11,12^

**Scheme 1:**
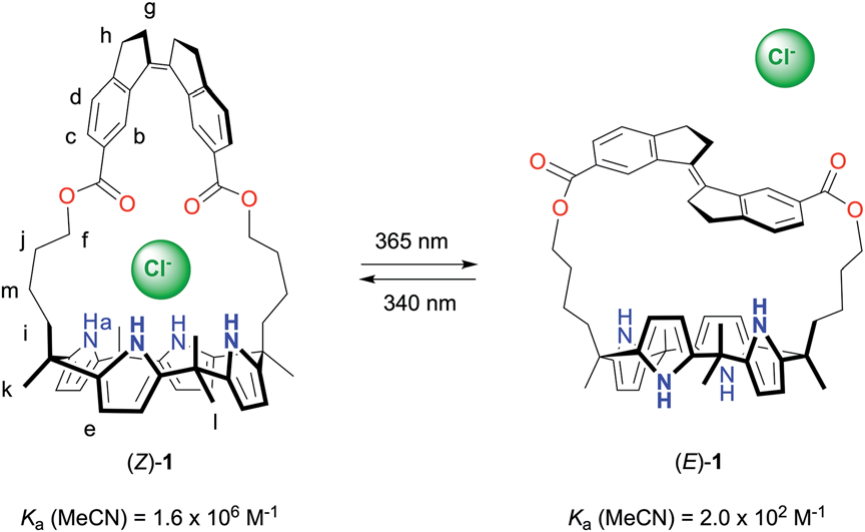
Photocontrolled chloride binding to receptor **1**.

Herein, we describe the synthesis and functioning of the stiff-stilbene strapped calix[4]pyrrole receptor **1** (Scheme 1). This receptor can be switched by light between *Z* and *E* forms, which display an extraordinary difference in affinity for chloride (8000-fold in MeCN). To the best of our knowledge, the largest differences in anion binding affinity reported for photoswitchable receptors up until now were much lower (around 10-to-100-fold).^7^ Hence, this work will likely stimulate the application of photoresponsive anion receptors as, for example, recyclable extractants or light-gated transporters.^7*e*,*p*^

## Results and discussion

### Calix[4]pyrrole receptor synthesis

**Scheme 2:**
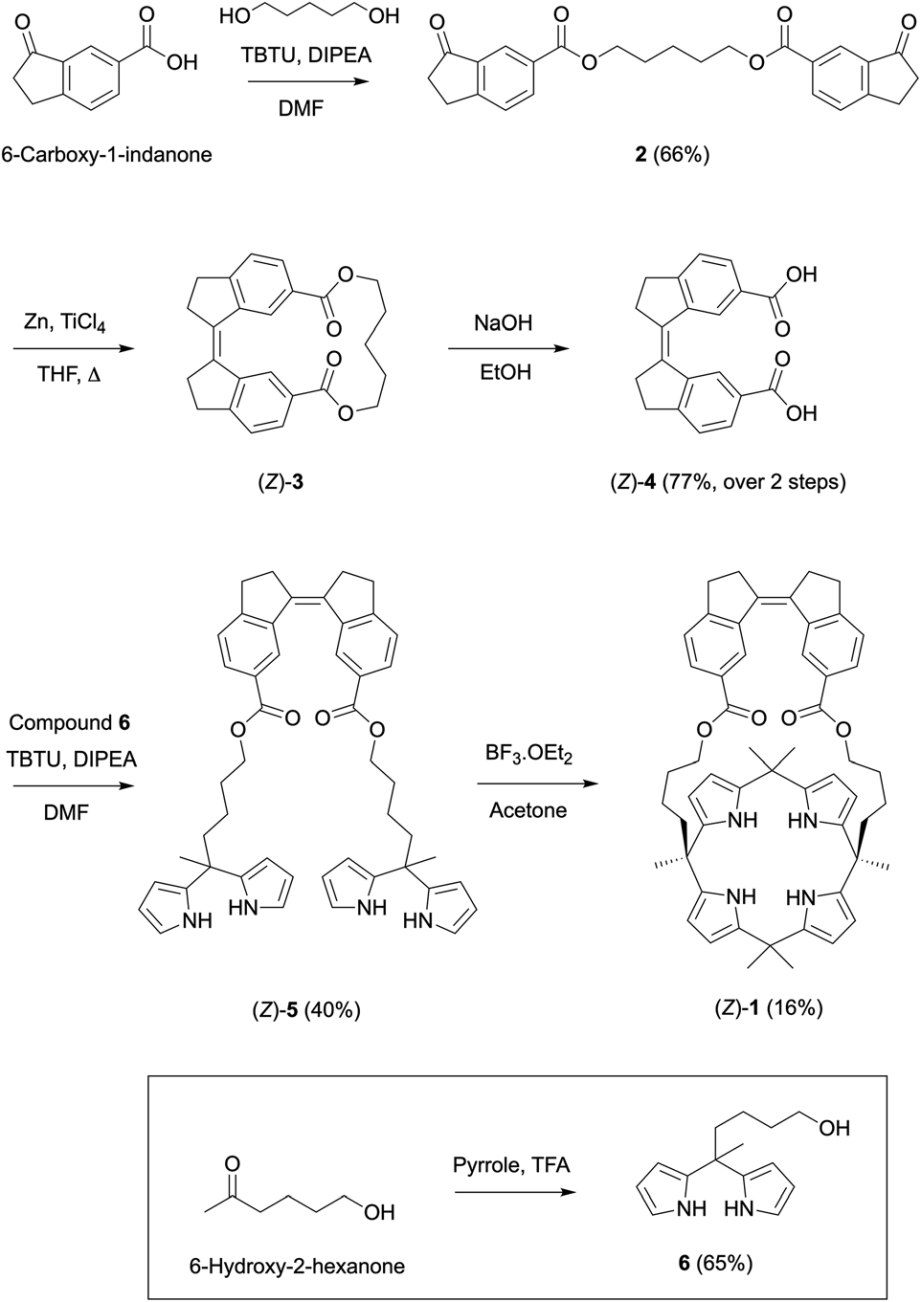
Synthesis of stiff-stilbene strapped calix[4]pyrrole receptor (*Z*)-**1**.

The synthesis of receptor (*Z*)-**1** is outlined in Scheme 2. The cyclic stiff-stilbene intermediate (*Z*)-**3** has been described earlier by Boulatov and co-workers^13^ and here, a slightly modified procedure was developed. That is, the reported 6-carboxy-1-indanone^14^ was coupled with 1,5-pentanediol in the presence of TBTU to give bis-indanone **2**. Subsequent intramolecular McMurry coupling afforded (*Z*)-**3**, which without purification was submitted to hydrolysis to obtain the bis-carboxylic acid derivative (*Z*)-**4**. In parallel, dipyrromethane **6** was synthesized by performing a ring opening of *δ*-valerolactone using a literature procedure,^15^ followed by condensation of the resulting 6-hydroxy-2-hexanone with pyrrole under acidic conditions. Then, dipyrromethane **6** and compound (*Z*)-**4** were coupled using TBTU to give bis-dipyrromethane (*Z*)-**5**, which was subsequently condensed with acetone in the presence of a catalytic amount of boron trifluoride to yield receptor (*Z*)-**1**. The expected structure was confirmed by NMR and MS analysis (see the ESI† for details).

### Photoswitching behavior

**Fig. 1:**
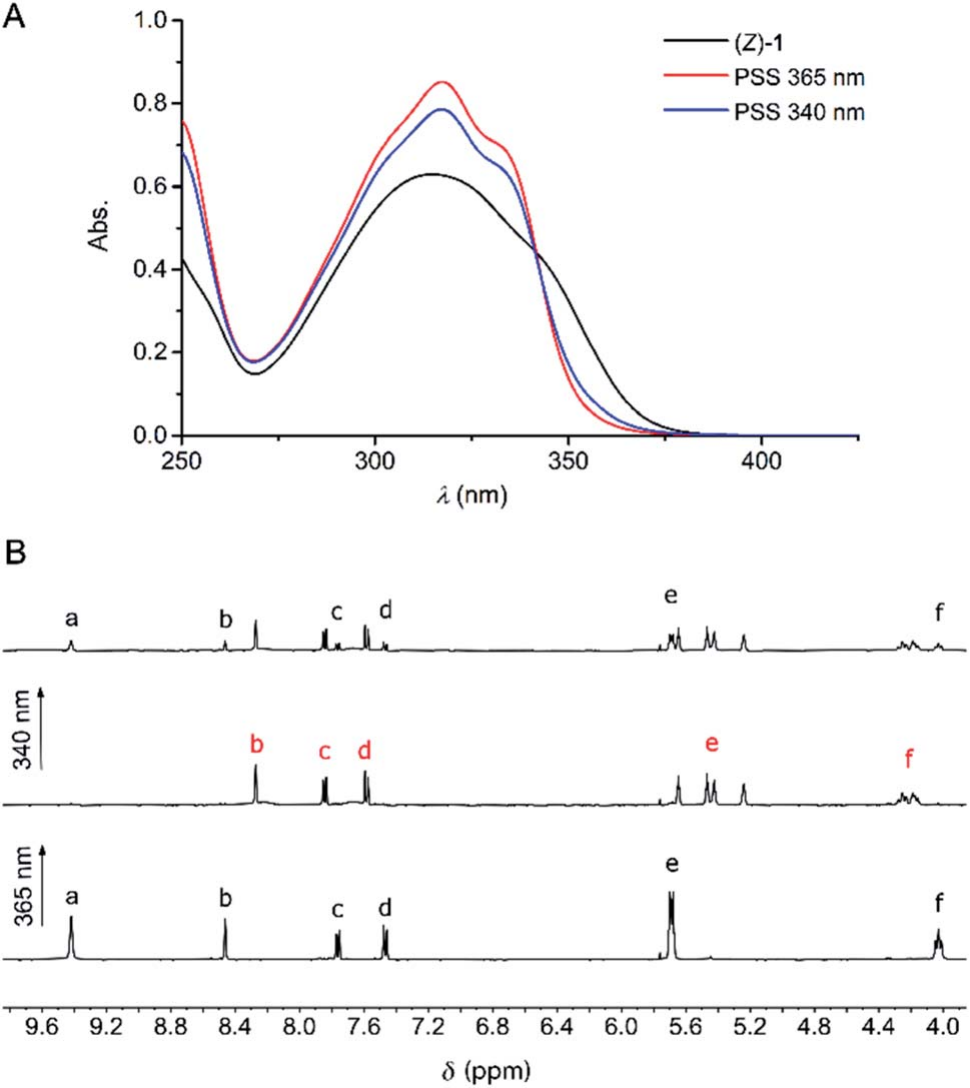
(A) UV-Vis spectral changes of (*Z*)-**1** (5 × 10^−4^ M in DMSO) upon consecutive 365 nm and 340 nm irradiation. (B) ^1^H NMR spectrum of (*Z*)-**1** (2 × 10^−3^ M in DMSO-*d*_6_) before (bottom) and after irradiation at 365 nm (middle) and 340 nm (top). For the lettering assignment, see Scheme 1.

The photoswitching properties of (*Z*)-**1** were first examined by UV-Vis spectroscopy in DMSO and MeCN. In both solvents, irradiation of (*Z*)-**1** with 365 nm light resulted in an increase of the absorption maximum (*λ*_max_ = 315 nm in DMSO and *λ*_max_ = 325 nm in MeCN) and a hypsochromic shift of the longest-wavelength absorption (Fig. 1A and S11–S12 in the ESI†), indicative of *Z* → *E* isomerization of stiff-stilbene.^11,12^ The opposite UV-Vis spectral changes were observed when the samples were subsequently irradiated with 340 nm light. In all cases, irradiation was continued until no further changes in absorption were noted, meaning that the photostationary states (PSS) had been reached.^16^

These irradiation processes were then monitored using ^1^H NMR spectroscopy by which the PSS ratios were determined. Exposure of (*Z*)-**1** in DMSO-*d*_6_ or MeCN-*d*_3_ to 365 nm light displayed significant ^1^H NMR spectral changes. In DMSO-*d*_6_ (Fig. 1B and S13 in the ESI†), the sharp singlet at *δ* = 9.42 ppm for the pyrrole NH protons (H_a_) disappeared and two broadened signals were observed further upfield (*δ* = 8.21 and 7.67 ppm). Furthermore, the multiplet at *δ* = 5.68 ppm for the β-pyrrolic protons (H_e_) splitted into four signals (between *δ* = 5.65 and 5.20 ppm) and the triplet at *δ* = 4.02 ppm for the aliphatic protons (H_f_) appeared as two downfield shifted multiplets (*δ* = 4.25 and 4.18 ppm). In addition, the aromatic signals belonging to the stiff-stilbene unit (H_b–d_) shifted from *δ* = 8.45, 7.76, and 7.47 ppm to *δ* = 8.27, 7.83 and 7.57 ppm, respectively, in line with *Z* → *E* isomerization.^11,12^ Importantly, this isomerization is nearly quantitative (>99%) in this case and the *Z*-isomer could be (partially) regenerated by irradiation with 340 nm light, resulting in the formation of a PSS mixture with a 27 : 73 (*Z*/*E*) ratio. No thermal isomerization of (*E*)-**1** back to (*Z*)-**1** was observed (Fig. S15 in the ESI†), which is in agreement with the high thermal isomerization barrier described for stiff-stilbene.^17^ Irradiation of (*Z*)-**1** in MeCN-*d*_3_ (Fig. S14 in the ESI†) led to very similar ^1^H NMR spectral changes, however, less broadening of the pyrrole NH signals (H_a_) was observed. The PSS_365_ and PSS_340_ ratios in this solvent were determined as 6 : 94 and 41 : 59 (*Z*/*E*), respectively.

### Single crystal X-ray crystallography

**Fig. 2:**
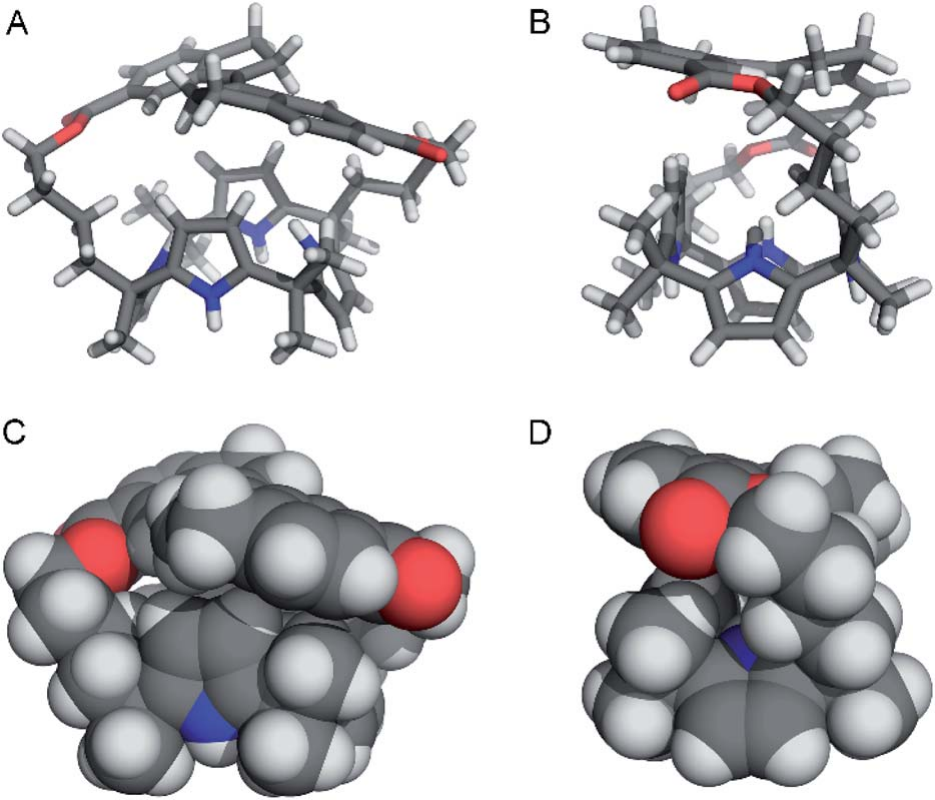
Front-view and side-view shown in stick (A and B) and CPK (C and D) representations of (*M*)–(*E*)-**1** found in the crystal structure. Only one of the enantiomers present in the unit cell is depicted and co-crystallized MeCN has been omitted for clarity.

Single crystals of (*E*)-**1** suitable for X-ray structure determination were obtained by slow evaporation of a solution in MeCN (see the ESI† for details). The solid state structure shown in Fig. 2 reveals a 1,3-alternate conformation which is also seen in other calix[4]pyrroles.^8,9^ Furthermore, the two alkyl chains of the strap are folded in slightly different ways and the stiff-stilbene moiety shows a deviation from its preferred planarity, indicating strain in the macrocycle (C_Ar_–C=C–C_Ar_ dihedral angle: 166.5°).^12*b*^ Interestingly, the two pyrrole groups that are the closest to the sideways oriented alkyl chains point with their nitrogen atoms toward the strap, while the other two point away from it, which minimizes steric interactions. In the crystal lattice, one of the pyrrolic NH atoms is hydrogen bonded to the ester moiety of a neighboring molecule (N–H⋯O distance: 2.95 Å) and furthermore, two enantiomers are present [*i.e.*, (*P*)–(*E*)-**1** and (*M*)–(*E*)-**1**].

### Chloride binding properties

**Fig. 3:**
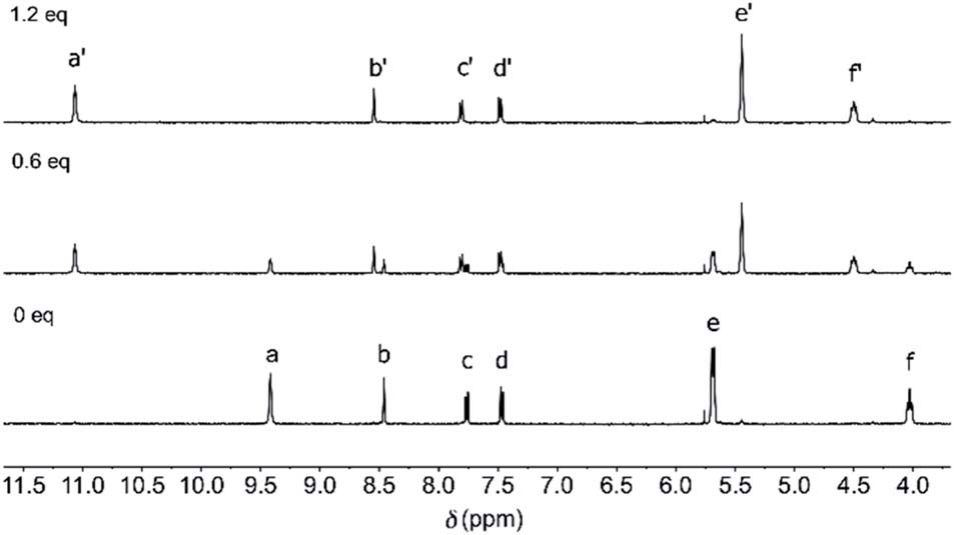
^1^H NMR spectrum of (*Z*)-**1** (2.4 mM in DMSO-*d*_6_) before (bottom) and after addition of 0.6 eq. (middle) and 1.2 eq. (top) of [NBu_4_]^+^[Cl]^–^. For the lettering assignment, see Scheme 1.

Chloride binding was studied in DMSO-*d*_6_ by ^1^H NMR spectroscopy. When 0.6 equivalent of tetrabutylammonium chloride ([NBu_4_]^+^[Cl]^–^) was added to (*Z*)-**1**, a new set of signals appeared alongside the original signals (Fig. 3 and S16 in the ESI†). This observation of two sets of signals is indicative of slow association/dissociation kinetics on the NMR timescale. The newly appeared signals for the pyrrole NH (H_a′_) and β-pyrrolic protons (H_e′_) were significantly shifted, as can be expected for chloride binding. Furthermore, the aliphatic protons (H_f′_) experienced a downfield shift, which hints at participation in binding and thus, chloride binding inside the central pocket defined by the strap (*vide infra*). Beyond addition of 1 equivalent of [NBu_4_]^+^[Cl]^–^, the original ^1^H NMR signals completely disappeared, indicative of full complexation.

On the contrary, when [NBu_4_]^+^[Cl]^–^ (0–50 eq.) was added to a solution of (*E*)-**1** in DMSO-*d*_6_, only very minor changes in chemical shifts were noted (Fig. S17 in the ESI†), revealing that almost no binding occurs at concentrations in the millimolar regime.^18^ Alternatively, when a solution of (*Z*)-**1** in the presence of an excess of chloride was irradiated with 365 nm light, the ^1^H NMR spectrum was virtually the same as for (*E*)-**1** with post-added chloride (Fig. S20 in the ESI†). This result suggests that chloride is expelled from the central binding pocket upon *Z* → *E* isomerization. Importantly, the formation of aggregates, that could interfere with binding, was excluded on the basis of ^1^H NMR dilution studies (Fig. S21 in the ESI†).

As no quantitative estimate of the chloride binding affinity could be made by ^1^H NMR spectroscopy, we turned to isothermal titration calorimetry (ITC).^9,19^ ITC measurements were performed in dry acetonitrile at 25 °C. The interaction between (*Z*)-**1** and [NBu_4_]^+^[Cl]^–^ appeared as a clean, exothermic 1 : 1 binding process (Fig. S22 in the ESI†), which was mainly driven by enthalpy (Δ*H* = −8.1 kcal mol^−1^), with only a small entropic contribution (Δ*S* = 1.1 cal mol^−1^). The affinity constant, determined as *K*_a_ = 1.6 × 10^6^ M^−1^, is within the range of values reported for phenyl-strapped calix[4]pyrrole receptors containing ester and ether linkers (*K*_a_ = 1.4–3.6 × 10^6^ M^−1^).^9*b*^ For (*E*)-**1**, on the other hand, under similar conditions only very minor heat release was produced and no binding parameters could be derived from these data (Fig. S22 in the ESI†).^18^

Nevertheless, an affinity constant for chloride binding to (*E*)-**1** could be obtained by a ^1^H NMR titration in MeCN-*d*_3_. Stepwise addition of [NBu_4_]^+^[Cl]^–^ led to gradual changes in chemical shifts and broadening of the signals for the pyrrole NH protons (H_a_) into the baseline (Fig. S18 in the ESI†). Similar broadening of the NH signals was observed earlier for [NBu_4_]^+^[Cl]^–^ addition to a phenanthroline-strapped calix[4]pyrrole and may be characteristic of a relatively weak anion-receptor interaction,^20^ and could tentatively be ascribed to on/off binding (as well as associated conformational changes) being intermediate on the NMR timescale. Hence, the titration data was analyzed using the ^1^H NMR chemical shift changes of the β-pyrrolic protons (H_e_) and the stiff-stilbene aromatic protons (H_b–d_). Simultaneous fitting of these data to a 1 : 1 binding model with HypNMR^21^ afforded an affinity constant of *K*_a_ = 2.0 × 10^2^ M^−1^ (Fig. S19 in the ESI†), which is 8000 times lower than the constant determined by ITC for chloride binding to (*Z*)-**1** (*vide supra*).^19^

### DFT-optimization of chloride complex

**Fig. 4:**
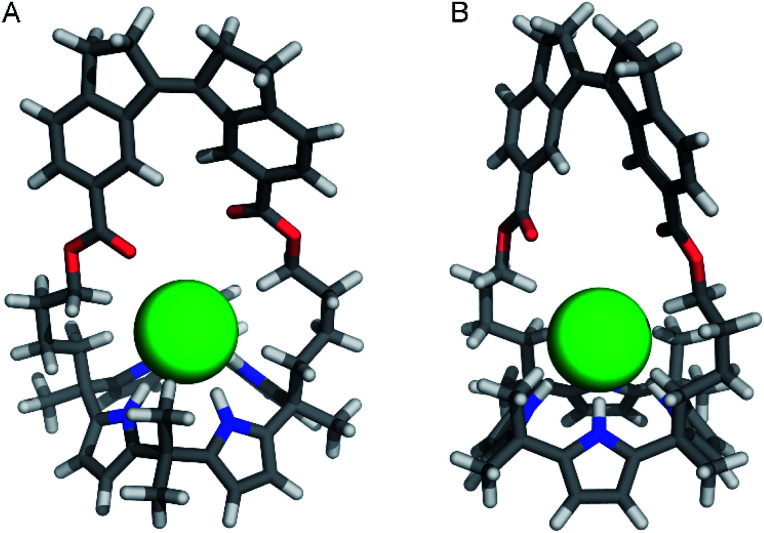
Front-view (A) and side-view (B) of the DFT-optimized geometry [B3LYP/6-31+G(d,p) method] of (*P*)–(*Z*)-**1** ⊂ Cl^–^.

Attempts to grow single crystals suitable for X-ray analysis of the *Z*-isomer in the presence of [NBu_4_]^+^[Cl]^–^ were unsuccessful and hence, the geometry of (*Z*)-**1** ⊂ Cl^–^ was optimized using DFT (see the ESI† for details). Different input structures were generated and optimized by the PM3 semi-empirical and the B3LYP/STO-3G methods first. The lowest energy structures were further optimized using the B3LYP/6-31+G (d,p) level of theory and an IEFPCM MeCN solvation model. In 1 : 1 complexes with chloride, calix[4]pyrrole has been found to adopt a cone conformation with all four pyrrole NH groups pointing toward the strap and participating in hydrogen bonding.^8,9^ The input structures of (*Z*)-**1** ⊂ Cl^–^ were prepared accordingly and the calculations revealed that chloride encapsulation is viable (Fig. 4 and Table S2 in the ESI†). Importantly, in the energy-minimized geometry, the average NH⋯Cl^–^ hydrogen bond distance (2.371 Å) is close to that observed in the solid state structure for phenyl-strapped calix[4]pyrrole (2.425 Å).^9*b*^ Furthermore, in line with observed ^1^H NMR chemical shift changes upon chloride addition (*vide supra*), CH⋯Cl^–^ interactions appear to be feasible for the methylene (3.835 Å for H_i_ and 3.040 Å for H_f_) and methyl protons (2.848 Å).

Similar computations for (*E*)-**1** ⊂ Cl^–^ also converged (Table S3 in the ESI†) and showed virtually the same average NH⋯Cl^–^ distance (2.370 Å). However, the stiff-stilbene strap drifted to one side during the DFT minimization, most likely as a result of the steric interactions with the chloride ion. Alternatively, a cone conformation with all NH atoms pointing away from the strap was considered, but did not converge at the higher DFT level. The latter conformation gives rise to unfavorable steric interactions between the β-pyrrolic protons and the strap. Hence, we tentatively assume that the weak chloride binding to (*E*)-**1** is a consequence of a lack of space in the central cavity provided by the strap as well as a high energy cost for adopting the cone conformation.

## Conclusions

In summary, we have successfully developed a calix[4]pyrrole receptor with a photomodulable strap by incorporating stiff-stilbene. In its *Z*-isomer, this receptor exhibits strong chloride binding similar to other strapped calix[4]pyrrole systems. When switched by light to the respective *E*-isomer, however, strain is built up in the strap and remarkably, chloride binding affinity is drastically reduced. In DMSO, this manifests itself in switching from a fully associated to a virtually fully dissociated state in the millimolar concentration regime. Quantification of the binding affinity in MeCN revealed an unparalleled 8000-fold difference between photoaddressable states. Such an enormous affinity difference opens new opportunities toward, for example, on demand substrate uptake and release in extractions and control of transport processes. Current efforts in our lab are focused on these themes, as well as on improving the photoswitching properties of stiff-stilbene.

## Experimental section

### Synthesis of calix[4]pyrrole receptor (*Z*)-**1**

BF_3_·OEt_2_ (10 μL) was added to compound (*Z*)-**5** (0.11 g, 0.15 mmol) in acetone (100 mL). The resulting mixture was stirred for 1 h at rt, after which triethylamine (0.3 mL) was added and the solvent was evaporated under reduced pressure. Purification by column chromatography (SiO_2_, CH_2_Cl_2_) yielded (*Z*)-**1** (20 mg, 16%) as a light brown solid. ^1^H NMR (400 MHz, DMSO-*d*_6_) *δ*_H_ 9.41 (s, 4H; H_a_), 8.46 (d, *J* = 1.6 Hz, 2H; H_b_), 7.76 (dd, *J* = 7.9, 1.5 Hz, 2H; H_c_), 7.47 (d, *J* = 7.9 Hz, 2H; H_d_), 5.68 (m, 8H; H_e_), 4.03 (t, *J* = 7.6 Hz, 4H; H_f_), 3.10–2.78 (m, 8H; H_g,h_), 1.96 (m, 4H; H_i_), 1.54 (m, 4H; H_j_), 1.49–1.40 (m, 18H; H_k,l_), 1.20 (m, 4H; H_m_); ^13^C NMR (400 MHz, CDCl_3_) *δ*_C_ 168.0, 153.8, 140.6, 138.6, 137.2, 135.3, 129.5, 128.5, 125.2, 124.5, 103.8, 103.3, 64.3, 40.5, 39.1, 35.5, 34.6, 30.9, 29.4, 28.5, 27.9, 21.7; HRMS (ESI) *m*/*z*: 829.4677 ([M + H]^+^, calcd for C_54_H_61_N_4_O_4_^+^: 829.4687).

## Author contributions

D. V. and S. J. W. conceived the project. D. V. synthesized the materials and carried out the photoisomerization and binding studies in solution. M. A. S. determined the single crystal X-ray structure. S. J. W. performed DFT calculations and guided the project. D. V. and S. J. W. wrote the manuscript.

## Conflicts of interest

There are no conflicts to declare.

## Supplementary Material

SC-012-D0SC06686A-s001

SC-012-D0SC06686A-s002

## References

[cit1] (a) Sessler J. L., Gale P. A. and Cho W. S., Anion Receptor Chemistry, ed. J. F. Stoddart, RSC, London, 2006

[cit2] Gunnlaugsson T., Glynn M., Tocci G. M., Kruger P. E., Pfeffer F. M. (2006). Coord. Chem. Rev..

[cit3] Moyer B. A., Delmau L. H., Fowler C. J., Ruas A., Bostick D. A., Sessler J. L., Katayev E., Pantos G. D., Llinares J. M., Hossain M. A., Kang S. O., Bowman-James K. (2006). Adv. Inorg. Chem..

[cit4] Valkenier H., Davis A. P. (2013). Acc. Chem. Res..

[cit5] Busschaert N., Caltagirone C., Van Rossom W., Gale P. A. (2015). Chem. Rev..

[cit6] Lee S., Flood A. H. (2013). J. Phys. Org. Chem..

[cit7] Wang Y., Bie F., Jiang H. (2010). Org. Lett..

[cit8] Gale P. A., Sessler J. L., Král V., Lynch V. (1996). J. Am. Chem. Soc..

[cit9] Yoon D.-W., Hwang H., Lee C.-H. (2002). Angew. Chem., Int. Ed..

[cit10] Peng S., He Q., Vargas-Zuñiga G. I., Qin L., Hwang I., Kim S. K., Heo N. J., Lee C.-H., Dutta R., Sessler J. L. (2020). Chem. Soc. Rev..

[cit11] Waldeck D. H. (1991). Chem. Rev..

[cit12] Shimasaki T., Kato S., Ideta K., Goto K., Shinmyozu T. (2007). J. Org. Chem..

[cit13] Akbulatov S., Tian Y., Boulatov R. (2012). J. Am. Chem. Soc..

[cit14] Horwell D. C., Howson W., Nolan W. P., Ratcliffe G. S., Ress D., Willems H. M. G. (1995). Tetrahedron.

[cit15] Ghosh A. K., Nicponski D. R. (2011). Org. Lett..

[cit16] An isosbestic point was maintained at *λ* = 345 nm in DMSO and at *λ* = 350 nm in MeCN throughout these irradiation processes, indicative of unimolecular conversion. After multiple switching cycles, a slight decay in absorption was noted (Fig. S11 and S12 in the ESI†)

[cit17] Kucharski T. J., Boulatov R. (2011). J. Mater. Chem..

[cit18] Please note that a highly concentrated sample of (*E*)-**1** to perform binding studies could not be prepared as it was obtained by irradiation of a sample of (*Z*)-**1**

[cit19] Sessler J. L., Gross D. E., Cho W. S., Lynch V. M., Schmidtchen F. P., Bates G. W., Light M. E., Gale P. A. (2006). J. Am. Chem. Soc..

[cit20] Heo N. J., Oh J. H., Lee J. T., He Q., Sessler J. L., Kim S. K. (2020). Org. Chem. Front..

[cit21] Frassineti C., Ghelli S., Gans P., Sabatini A., Moruzzi M. S., Vacca A. (1995). Anal. Biochem..

