# Supplementary material for: A photoswitchable strapped calix[4]pyrrole receptor: highly effective chloride binding and release
Source: Chem Sci. 2021 Jan 13;12(9):3188–93. doi: 10.1039/d0sc06686a (PMC8179391; doi:10.1039/d0sc06686a)
Supplement: SC-012-D0SC06686A-s001 [file SC-012-D0SC06686A-s001.pdf]

Electronic Supplementary Material (ESI) for Chemical Science.
This journal is © The Royal Society of Chemistry 2021

ELECTRONIC SUPPLEMENTARY INFORMATION (ESI)

A Photoswitchable Strapped Calix[4]pyrrole Receptor: Highly Effective Chloride Binding and Release

David Villarón,[a] Maxime A. Siegler,[b] and Sander J. Wezenberg*[a]

[a] *Leiden Institute of Chemistry, Leiden University, Einsteinweg 55, 2333 CC Leiden (The Netherlands)*

[b] *Department of Chemistry, Johns Hopkins University 3400 N. Charles St., Baltimore, MD, 21218 (United States)*

Email: s.j.wezenberg@lic.leidenuniv.nl

Table of Contents

Experimental section 2
^{1}H and ^{13}C NMR Spectra of new compounds 6
UV-Vis photoisomerization studies in DMSO 16
UV-Vis photoisomerization studies in MeCN 17
^{1}H NMR photoisomerization studies 18
^{1}H NMR thermal stability studies of (*E*)-**1** 19
^{1}H NMR titration experiment of (*Z*)-**1** 20
^{1}H NMR titration experiment of (*E*)-**1** 21
Titration curve analysis 23
^{1}H NMR photoisomerization of (*Z*)-**1**$\subset$Cl$^-$ 24
NMR dilution experiment of (*E*)-**1** 25
ITC titration experiments 26
Single crystal X-ray crystallography 27
Geometry optimization by DFT 29
References 36

Experimental section

General methods and materials:

THF, MeCN, and Et_2O were dried using a Pure Solve 400 solvent purification system from Innovative Technology. Dry DMSO and DMF were purchased from Acros Organics and DMSO-d_6 and $CDCl_3$, were purchased from Eurisotop. DMSO-d_6 was stored under N_2 over molecular sieves (4Å). The degassing of the solvents was carried out by purging with N_2 for 30 min. 6-Carboxy-1-indanone[1] and 6-Hydroxy-2-hexanone[2] were prepared according to procedures reported in the literature. All other chemicals were commercial products and were used without further purification. Column chromatography was performed using silica gel (SiO_2) purchased from Screening Devices BV (Pore diameter 55-70 Å, surface area 500 m^2g^{-1}) and thin-layer chromatography (TLC) was carried out on aluminum sheets coated with silica 60 F254 obtained from Merck. Compounds were visualized with UV light (254 nm) or by staining with potassium permanganate. ^1H and ^{13}C NMR spectra were recorded on Bruker AV 400 and Bruker 500 Ultra Shield instruments at 298 K unless indicated otherwise. Chemical shifts (δ) are denoted in parts per million (ppm) relative to residual protiated solvent (DMSO-d_6: for ^1H detection, δ = 2.50 ppm; for ^{13}C detection, δ = 39.52 ppm; $CDCl_3$: for ^1H detection, δ = 7.26 ppm; for ^{13}C detection, δ = 77.16 ppm. The splitting pattern of peaks is designated as follows: s (singlet), d (doublet), t (triplet), q (quartet), p (quintet), h (septet), m (multiplet), br (broad). High-resolution mass spectrometry (ESI-MS) was performed on a Thermo Scientific Q Exactive HF spectrometer with ESI ionization. UV-Vis spectra were recorded on an Agilent Cary 8454 spectrometer in a 1 cm or 1 mm quartz cuvette. ITC measurements were performed using a MicroCal VP-ITC MicroCalorimeter. Irradiation of UV-Vis and NMR samples was carried out using a Thorlab model M340F3 LED (0.85 mW) and a Thorlab model M365F1 LED (3.00 mW), positioned at a distance of 1 cm to the sample.

Pentane-1,5-diyl bis(3-oxo-2,3-dihydro-1*H*-indene-5-carboxylate (2):

In an oven dried flask, 6-carboxy-1-indanone (1.77 g, 10.0 mmol), 2-(1*H*-benzotriazole-1-yl)-1,1,3,3-tetramethylaminium tetrafluoroborate (TBTU) (3.20 g, 9.97 mmol), and DIPEA (3.5 mL, 20 mmol) were dissolved in dry DMF (27 mL) and the mixture was stirred for 30 min at rt. Then, 1,5-pentanediol (0.52 mL, 5.0 mmol) in dry DMF (4.4 mL) was added and the reaction mixture was further stirred for 14 h at rt. The mixture was then diluted with CH_2Cl_2 (20 mL) and washed with a 0.5 M aqueous HCl solution (3 x 20 mL), a 1M aqueous $NaHCO_3$ solution

(2 x 20 mL), and Brine (2 x 20 mL). The organic phase was dried over Na_2SO_4 and concentrated. Purification by column chromatography (SiO_2, pentane/EtOAc 1:1) afforded compound **2** (1.40 g, 66%) as a pale yellow solid. ^{1}H NMR (400 MHz, $CDCl_3$): 8.41 (s, 2H), 8.27 (dd, *J* = 8.0, 1.7 Hz, 2H), 7.56 (d, *J* = 8.0 Hz, 2H), 4.37 (t, *J* = 6.5 Hz, 4H), 3.21 (m, 4H), 2.76 (m, 4H), 1.87 (m, 4H), 1.62 (m, 2H); ^{13}C NMR (400 MHz, $CDCl_3$): 206.1, 165.9, 159.6, 137.3, 135.4, 130.0, 127.0, 125.2, 65.2, 36.5, 28.4, 26.1, 22.7; HRMS (ESI) *m/z:* 421.1642 ($[M+H]^+$, calcd for $C_{25}H_{25}O_6^+$: 421.1645).

(*Z*)-1,2,9,10,11,12,18,19-)Octahydro-8*H*-3,5:15,17-diethenodicyclopenta[k,m][1,7]dioxa-cycloheptadecine-6,14-dione [(*Z*)-3]:

A similar procedure to the one reported by Boulatov and co-workers was used.[3] $TiCl_4$ (0.98 mL, 8.9 mmol) was added dropwise to a suspension of zinc powder (1.16 g, 17.9 mmol) in dry THF (370 mL). The resulting mixture was stirred under reflux for 1.5 h and subsequently, compound **2** (0.75 g, 1.8 mmol) in dry THF (30 mL) was added to the refluxing mixture over 3 h by syringe pump. After the addition was completed, the mixture was stirred under reflux for a further 1.5 h. The mixture was then allowed to cool to rt, treated with a saturated aqueous NH_4Cl solution and extracted with EtOAc (3 x 30 mL). The combined organic layers were dried over Na_2SO_4 and concentrated to afford (*Z*)-**3** (yield n.d.) as a yellow oil, which was submitted to the next reaction step without further purification. NMR spectroscopic data was identical to that previously reported.[3]

(*Z*)-2,2',3,3'-Tetrahydro-[1,1'-biindenylidene]-6,6'-dicarboxylic acid [(*Z*)-4]:

The crude compound (*Z*)-**3** from the previous reaction was dissolved in EtOH (20 mL) and added to a basic solution of NaOH (1.15 g, 28.8 mmol) in EtOH (50 mL). The resulting mixture was stirred for 6 h, after which the mixture was concentrated. The residue was redissolved in water (40 mL) and addition of a 6M aqueous HCl solution (40 mL) led to the formation of a precipitate. The precipitate was filtered off, washed with water, and air-dried to afford (*Z*)-**4** (0.44 g, 77%, over 2 steps) as a dark red solid. ^{1}H NMR (400 MHz, DMSO-d_6): 12.78 (br s, 2H), 8.56 (s, 2H), 7.80 (dd, *J* = 7.9, 1.3 Hz, 2H), 7.46 (d, *J* = 7.9 Hz, 2H), 3.09-2.76 (m, 8H); ^{13}C NMR (400 MHz, DMSO-d_6): 167.2, 153.3 140.0, 134.5, 128.8, 128.6, 125.5, 123.4, 34.3, 30.2; HRMS (ESI) *m/z:* 321.1121 ($[M+H]^+$, calcd for $C_{20}H_{17}O_4^+$: 321.1120).

Bis(5,5-di(1*H*-pyrrol-2-yl)hexyl) (*Z*)-2,2',3,3'-tetrahydro-[1,1'-biindenylidene]-6,6'-dicarboxylate [(*Z*)-5]:

Compound (Z)-**4** (0.35 g, 1.09 mmol), TBTU (0.70 g, 2.2 mmol), and DIPEA (0.76 mL, 4.4 mmol) were dissolved in dry DMF (2.6 mL) and stirred for 30 min at rt. Then, compound **6** (0.63 g, 2.7 mmol) in dry DMF (2 mL) was added and the resulting mixture which was stirred for 3 days at rt. The mixture was diluted with CH_2Cl_2 (10 mL) and washed with a 0.5M aqueous HCl solution (2 x 10 mL), a saturated aqueous $NaHCO_3$ solution (2 x 10 mL), and Brine (10 mL). The organic phase was dried over Na_2SO_4 and concentrated. Purification by column chromatography (SiO_2, pentane/EtOAc 6:4) afforded (Z)-**5** (0.33 g, 40%) as a bright orange solid. ^{1}H NMR (400 MHz, DMSO-d_6): 10.17 (s, 4H), 8.55 (s, 2H), 7.75 (dd, *J* = 7.9, 1.5 Hz, 2H), 7.47 (d, *J* = 7.9 Hz, 2H), 6.55-6.49 (m, 4H), 5.84-5.78 (m, 4H), 5.66-5.60 (m, 4H), 4.11 (t, *J* = 6.4 Hz, 4H), 3.08-2.77 (m, 8H), 1.90 (m, 4H), 1.52 (m, 4H), 1.40 (s, 6H), 1.11 (m, 4H); ^{13}C NMR (400 MHz, DMSO-d_6): 165.6, 153.8, 134.0, 138.3, 134.9, 128.6, 127.7, 125.7, 123.2, 116.3, 106.2, 103.6, 64.3, 34.2, 30.2, 28.4, 24.8, 20.4; HRMS (ESI) *m/z:* 749.4061 ($[M+H]^+$, calcd for $C_{48}H_{52}N_4O_4^+$: 749.4061).

5,5-Di(1*H*-pyrrol-2-yl)hexan-1-ol (6):

6-Hydroxy-2-hexanone (1.60 g, 13.8 mmol) was dissolved in freshly distilled pyrrole (21 mL, 0.30 mol) and the solution was stirred at rt for 5 min, after which TFA (0.10 mL, 1.4 mmol) was added. The resulting mixture was stirred further for 10 min at rt and then a 0.1M aqueous NaOH solution (40 mL) was added. The aqueous phase was extracted with CH_2Cl_2 (3 x 30 mL) and the combined organic layers were dried over Na_2SO_4 and concentrated. Purification by column chromatography (SiO_2, CH_2Cl_2/EtOAc 7:3) yielded compound **6** (2.1 g, 65%) as a yellow oil. ^{1}H NMR (400 MHz, DMSO-d_6): 10.23 (s, 2H), 6.55 (m, 2H), 5.83 (m, 2H), 5.69 (m, 2H), 4.32 (m, 1H); 3.32 (m, 2H), 1.91 (m, 2H), 1.49 (s, 3H), 1.36 (p, *J* = 7.5 Hz, 2H), 1.07 (m, 2H); ^{13}C NMR (400 MHz, DMSO-d_6): 138.6, 116.3, 106.2, 103.6, 60.9, 54.9, 33.2, 25.0, 20.9, one signal must be hidden under the DMSO solvent signal; HRMS (ESI) *m/z:* 233.1646 ($[M+H]^+$, calcd for $C_{14}H_{20}N_2O^+$: 233.1648).

Stiff-stilbene strapped calix[4]pyrrole receptor (*Z*)-1:

$BF_3.OEt_2$ (10 µL) was added to compound (*Z*)-**5** (0.11g, 0.15 mmol) in acetone (100 mL). The resulting mixture was stirred for 1 h at rt, after which triethylamine (0.3 mL) was added and the solvent was evaporated under reduced pressure. Purification by column chromatography (SiO_2, CH_2Cl_2) yielded (Z)-**1** (20 mg, 16%) as a light brown solid. ^1H NMR (400 MHz, DMSO-d_6): 9.41 (s, 4H), 8.46 (d, *J* = 1.6 Hz, 2H), 7.76 (dd, *J* = 7.9, 1.5 Hz, 2H), 7.47 (d, *J* = 7.9 Hz, 2H), 5.68 (m, 8H), 4.03 (t, *J* = 7.3 Hz, 4H), 3.10-2.78 (m, 8H), 1.96 (t, *J* = 7.2 Hz, 4H), 1.54 (m, 4H), 1.49-1.40 (m, 18H), 1.20 (m, 4H); ^{13}C NMR (400 MHz, $CDCl_3$): 168.0, 153.8, 140.6, 138.6, 137.2, 135.3, 129.5, 128.5, 125.2, 124.5, 103.8, 103.3, 64.3, 40.5, 39.1, 35.5, 34.6, 30.9, 29.4, 28.5, 27.9, 21.7; HRMS (ESI) *m/z:* 829.4677 ($[M+H]^+$, calcd for $C_{54}H_{61}N_4O_4^+$: 829.4687).

^{1}H and ^{13}C NMR Spectra of new compounds

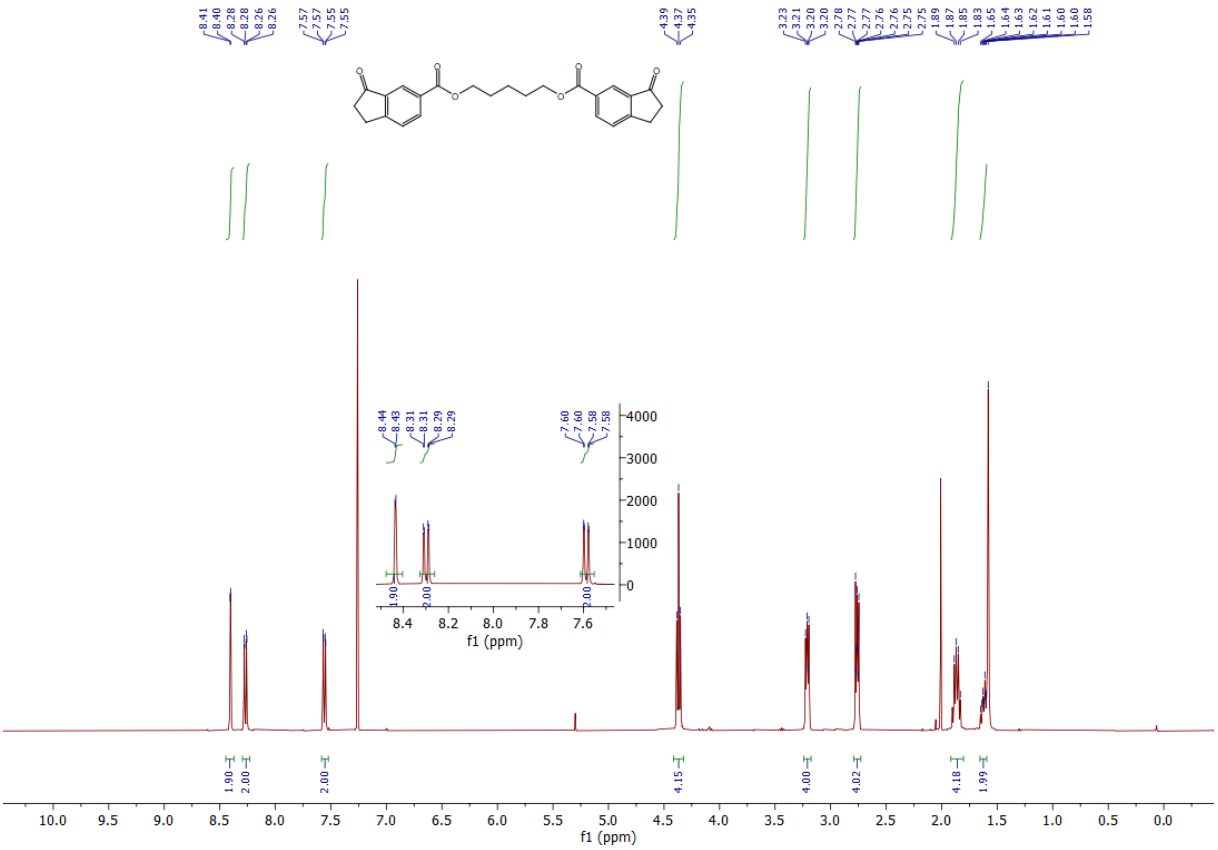

Figure S1. 400 MHz ^{1}H NMR spectrum of **2** measured at 298 K in $CDCl_3$.

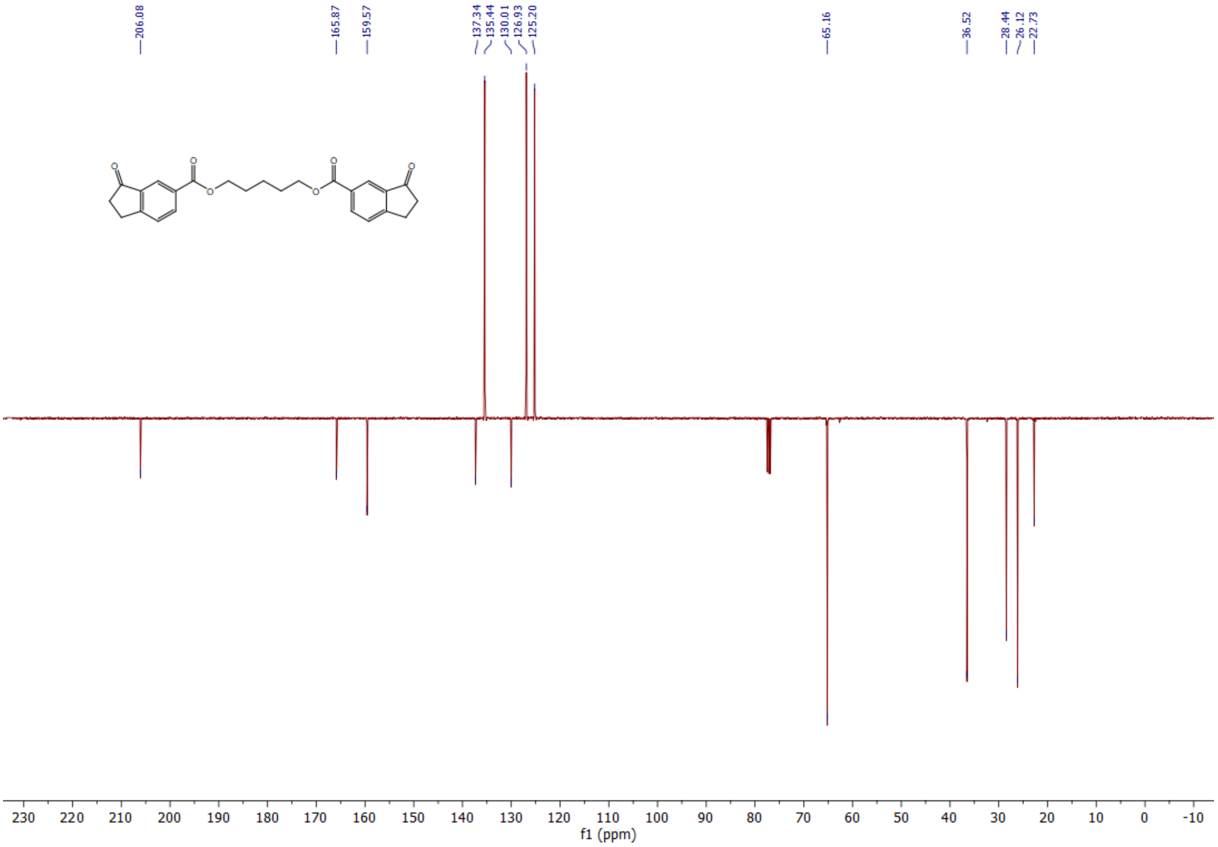

Figure S2. 400 MHz ^{13}C NMR APT spectrum of **2** measured at 298 K in $CDCl_3$: CH and CH_3 signals positive and quaternary carbon and CH_2 signals negative.

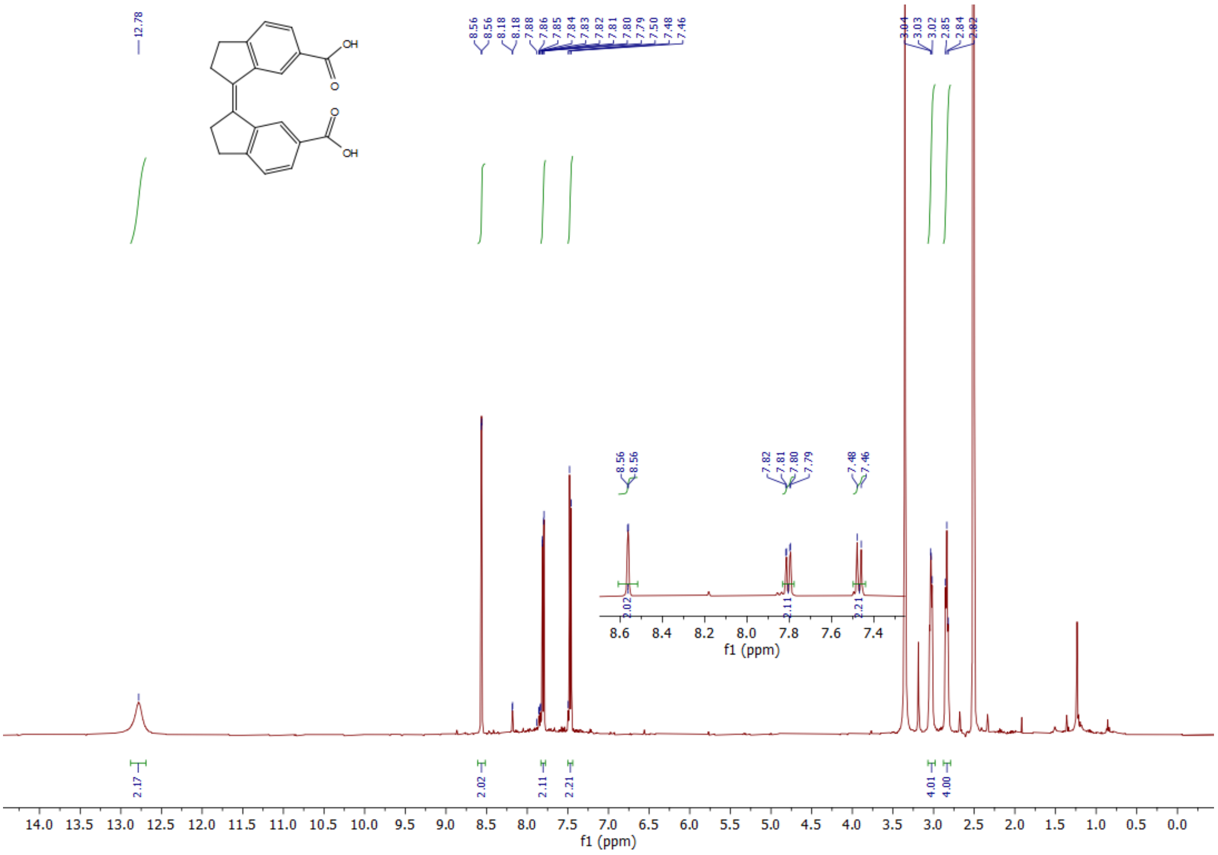

Figure S3. 400 MHz ¹H NMR spectrum of (*Z*)-**4** measured at 298 K in DMSO-d_6.

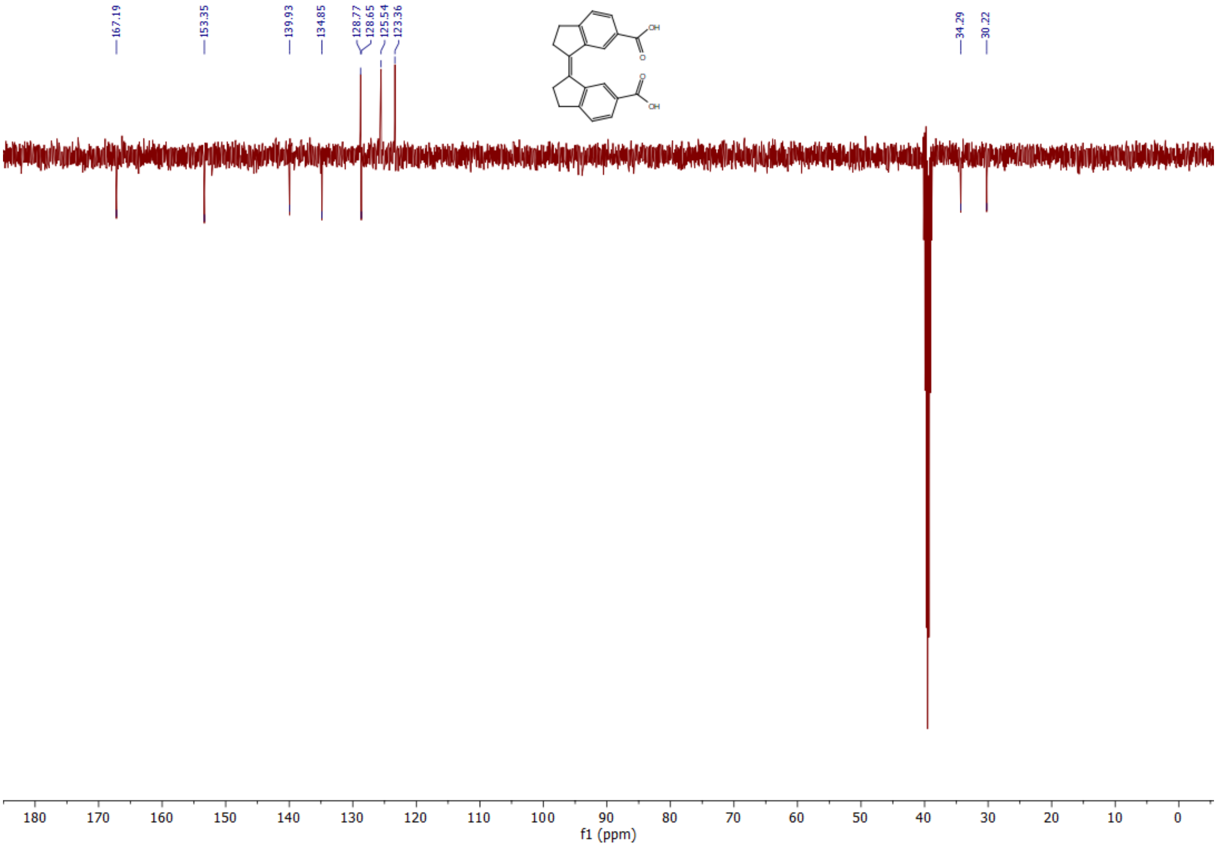

Figure S4. 400 MHz ^{13}C NMR APT spectrum of (Z)-**4** measured at 298 K in DMSO-d_6: CH and CH_3 signals positive and quaternary carbon and CH_2 signals negative.

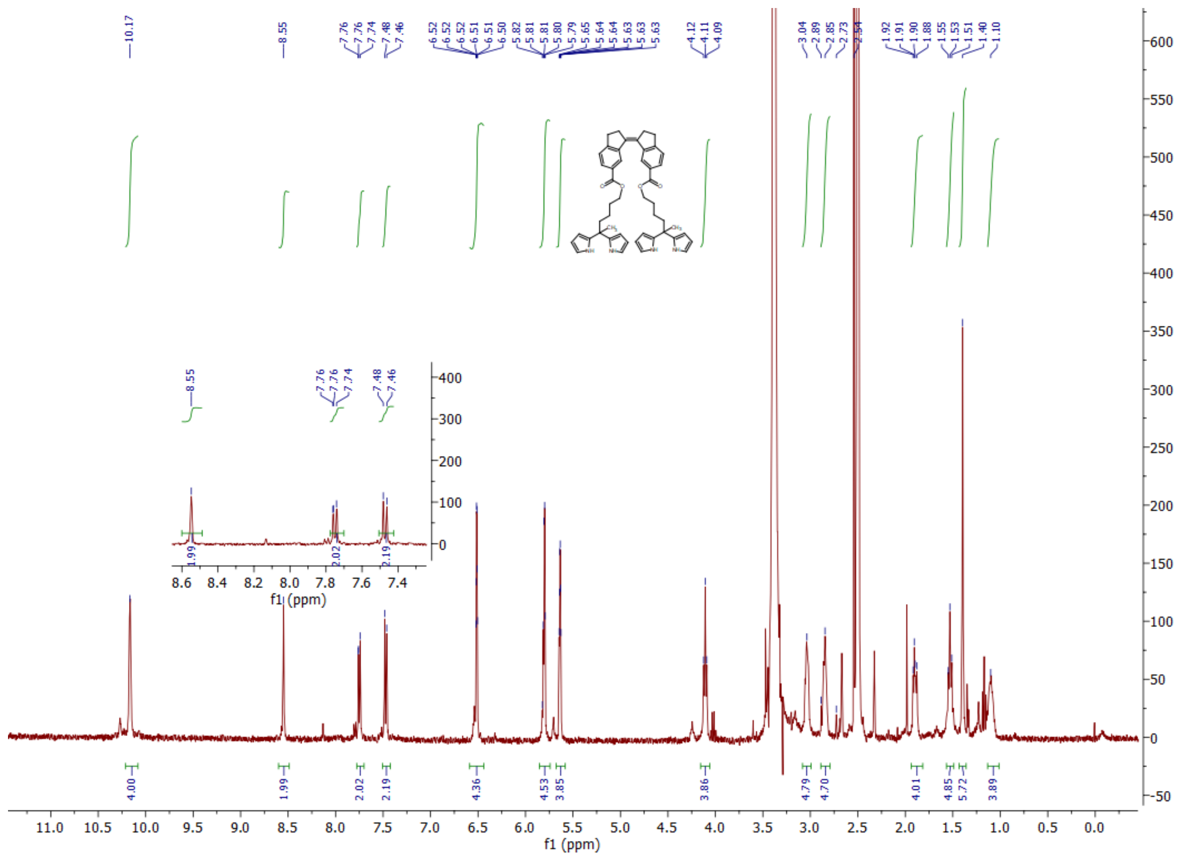

Figure S5. 400 MHz ^{1}H NMR spectrum of (Z)-**5** measured at 298 K in DMSO-d_6. The ^{1}H NMR signals of the minor impurity that is present belong to dipyrromethane **6**.

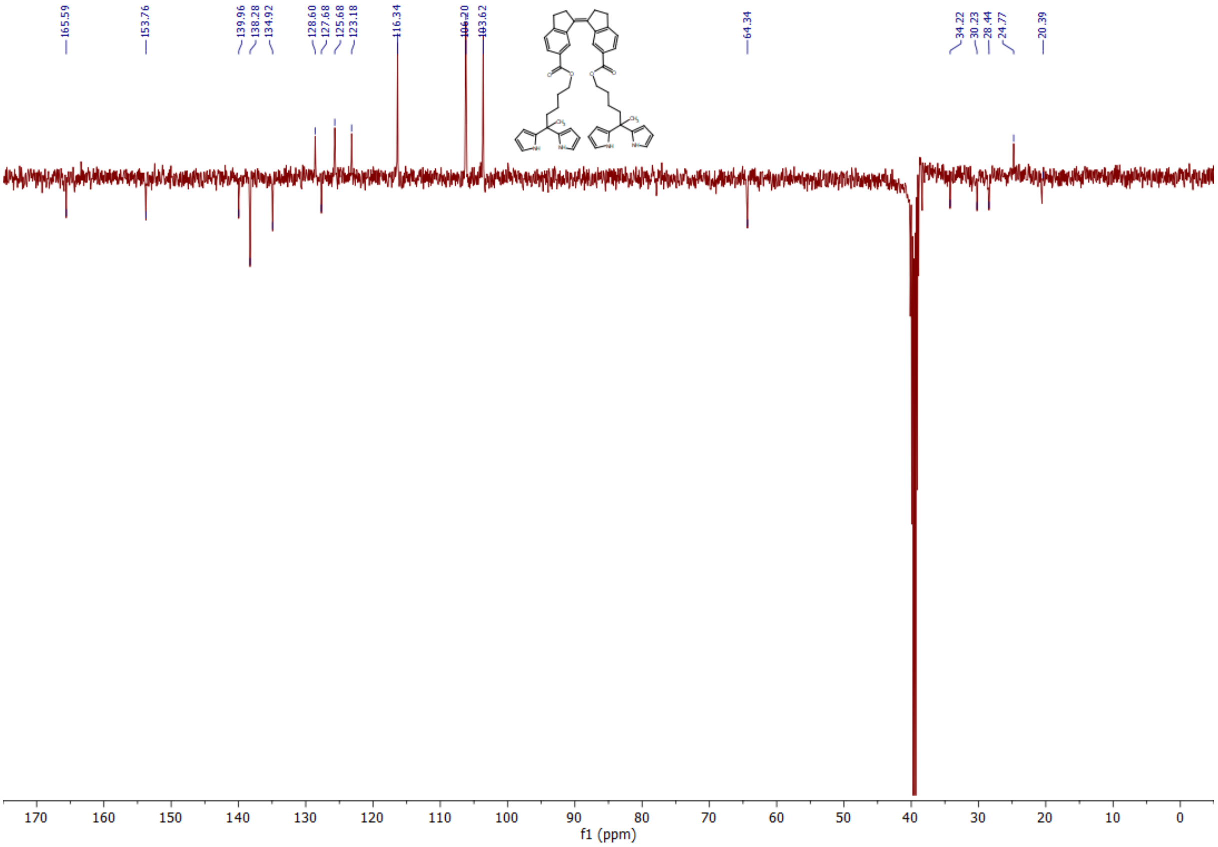

Figure S6. 400 MHz ^{13}C NMR APT spectrum of (*Z*)-**5** measured at 298 K in DMSO-d_6: CH and CH_3 signals positive and quaternary carbon and CH_2 signals negative.

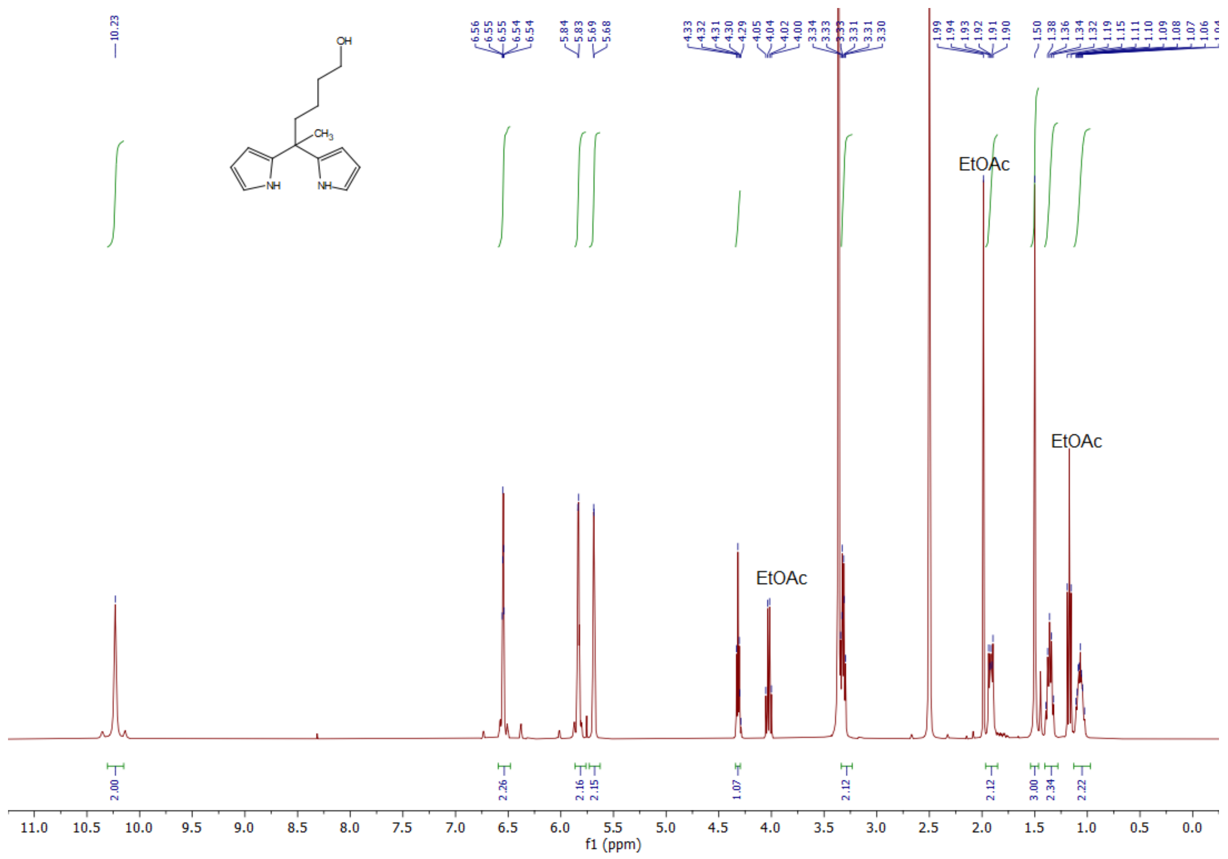

Figure S7. 400 MHz ^{1}H NMR spectrum of **6** measured at 298 K in DMSO-d_6.

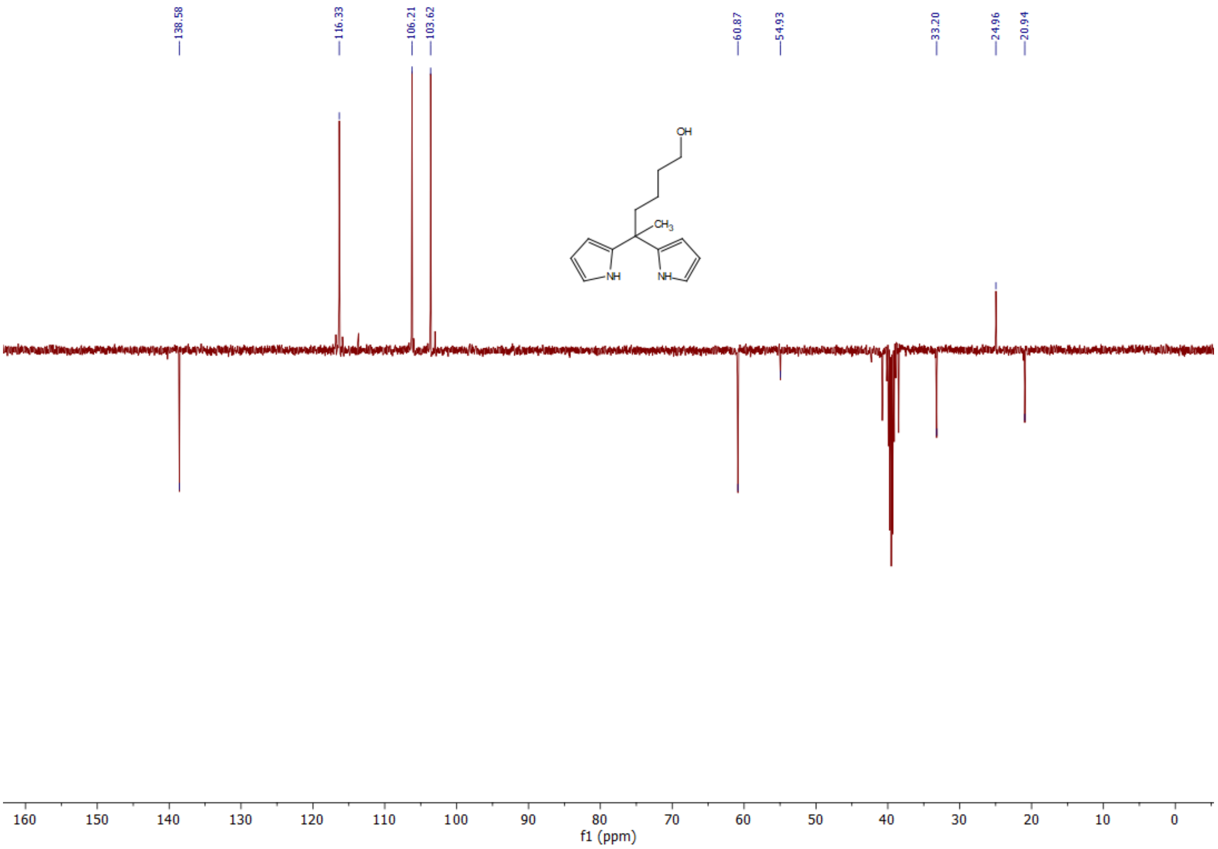

Figure S8.400 MHz ^{13}C NMR APT spectrum of **6** measured at 298 K in DMSO-d_6: CH and CH_3 signals positive and quaternary carbon and CH_2 signals negative.

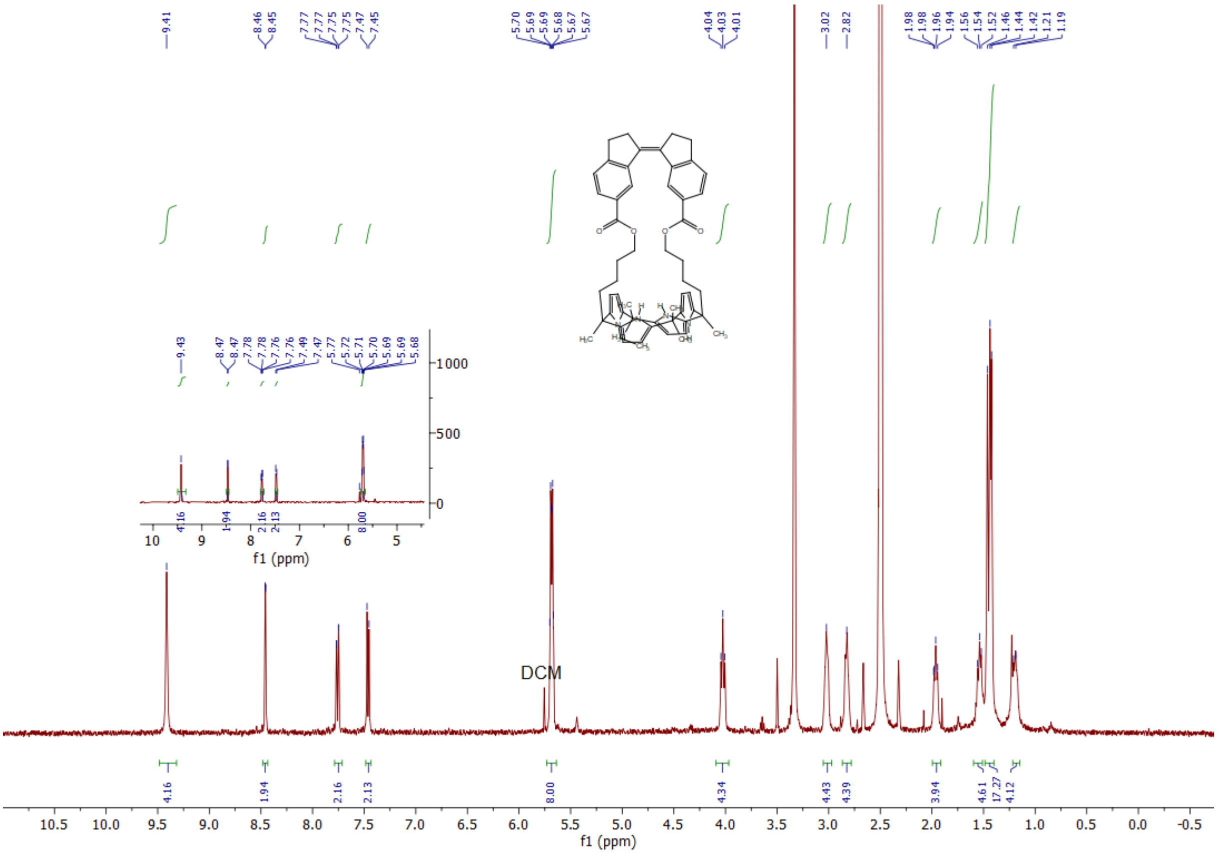

Figure S9. 400 MHz ^{1}H NMR spectrum of (*Z*)-**1** measured at 298 K in DMSO-d_6.

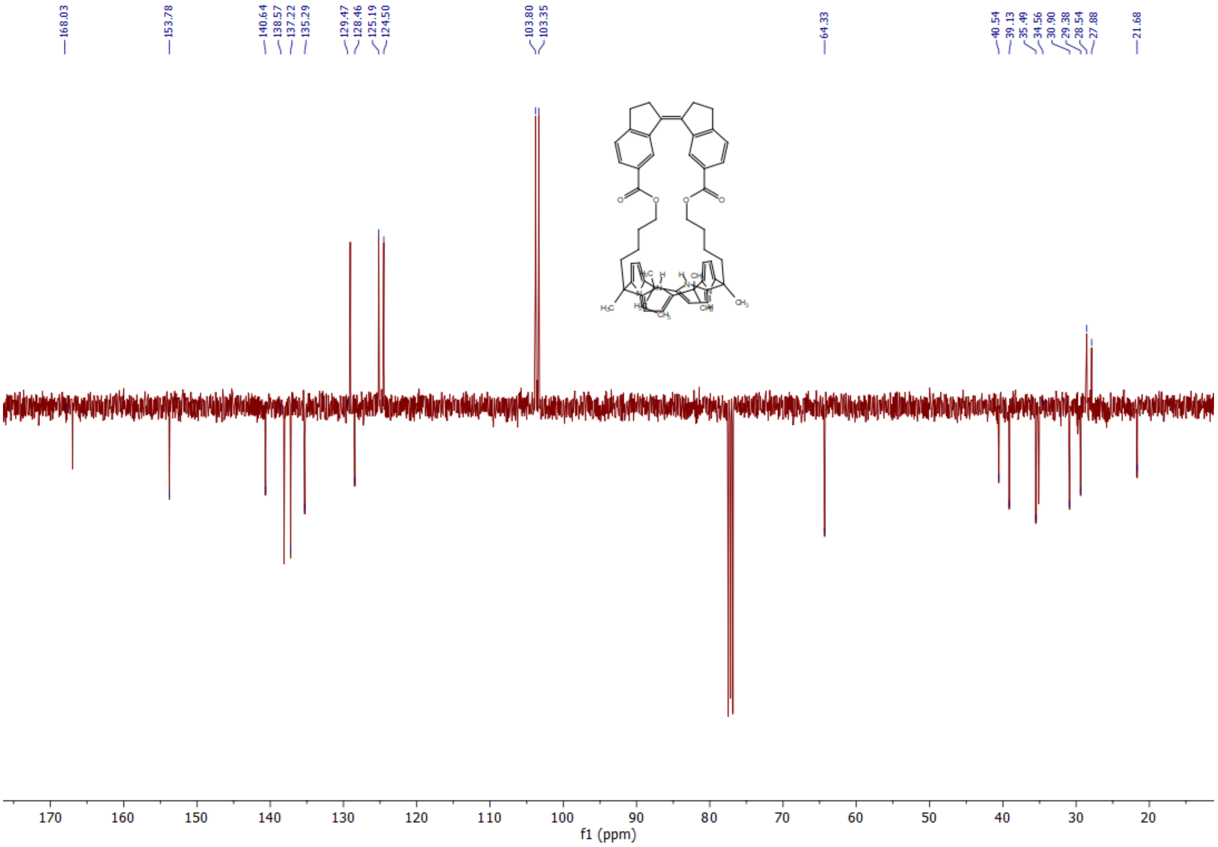

Figure S10. 400 MHz ^{13}C NMR APT spectrum of (*Z*)-**1** measured at 298 K in $CDCl_3$: CH and CH_3 signals positive and quaternary carbon and CH_2 signals negative.

UV-Vis photoisomerization studies in DMSO

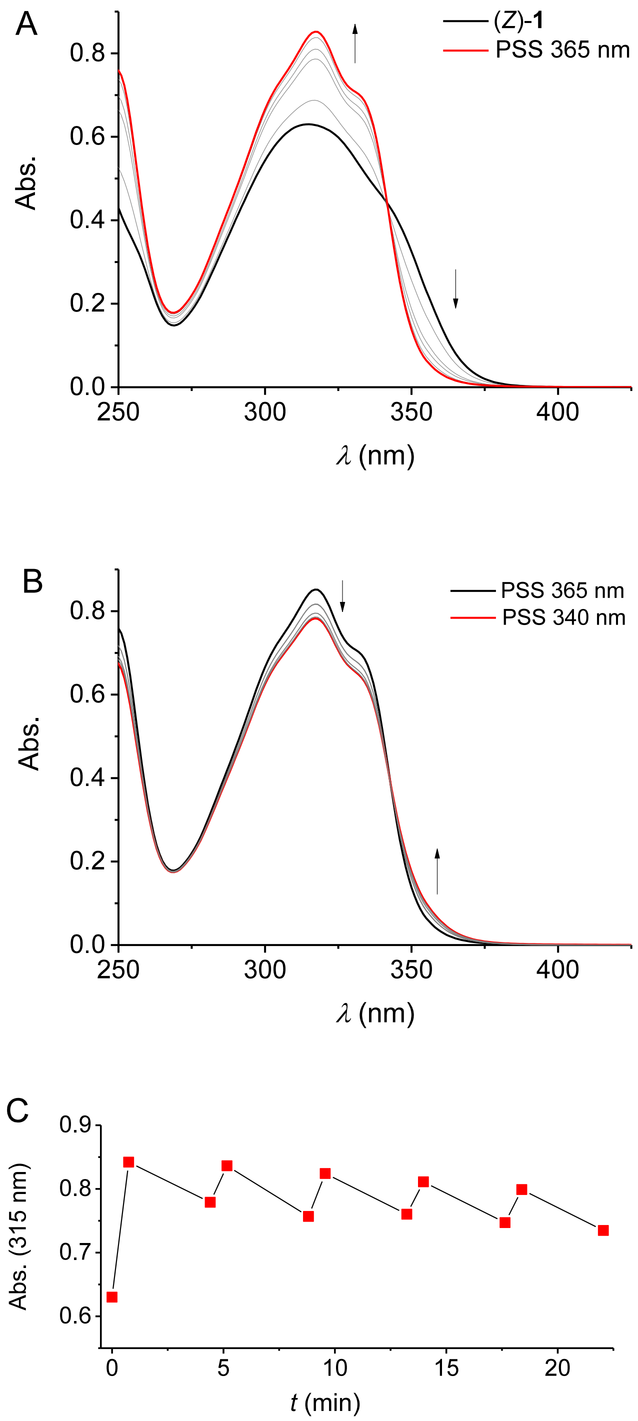

Figure S11. UV-Vis spectral changes of **1** (5×10^{-4} M in DMSO) upon (A) 365 nm irradiation for 45s and (B) 340 nm irradiation for 220s and (C) plot of the change in absorption at λ = 315 nm upon multiple 365/340 nm switching cycles. The overall decay in absorption after five switching cycles is 5.1% and 5.6 % after 365 nm and 340 nm irradiation, respectively.

UV-Vis photoisomerization studies in MeCN

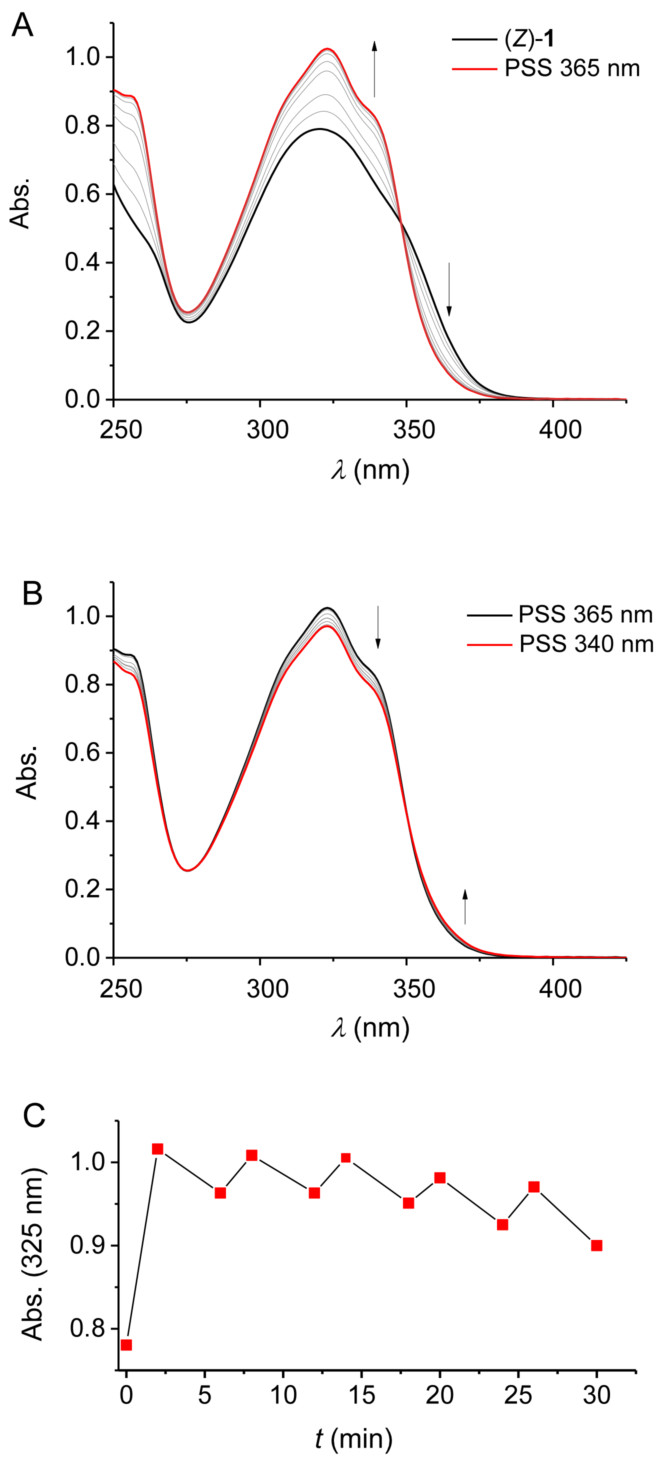

Figure S12. UV-Vis spectral changes of **1** (5×10^{-4} M in MeCN) upon (A) 365 nm irradiation for 100s and (B) 340 nm irradiation for 240s and (C) plot of the change in absorption at $\lambda = 325$ nm upon multiple 365/340 nm switching cycles. The overall decay in absorption after five switching cycles is 4.3% and 6.8 % after 365 nm and 340 nm irradiation, respectively.

^{1}H NMR photoisomerization studies

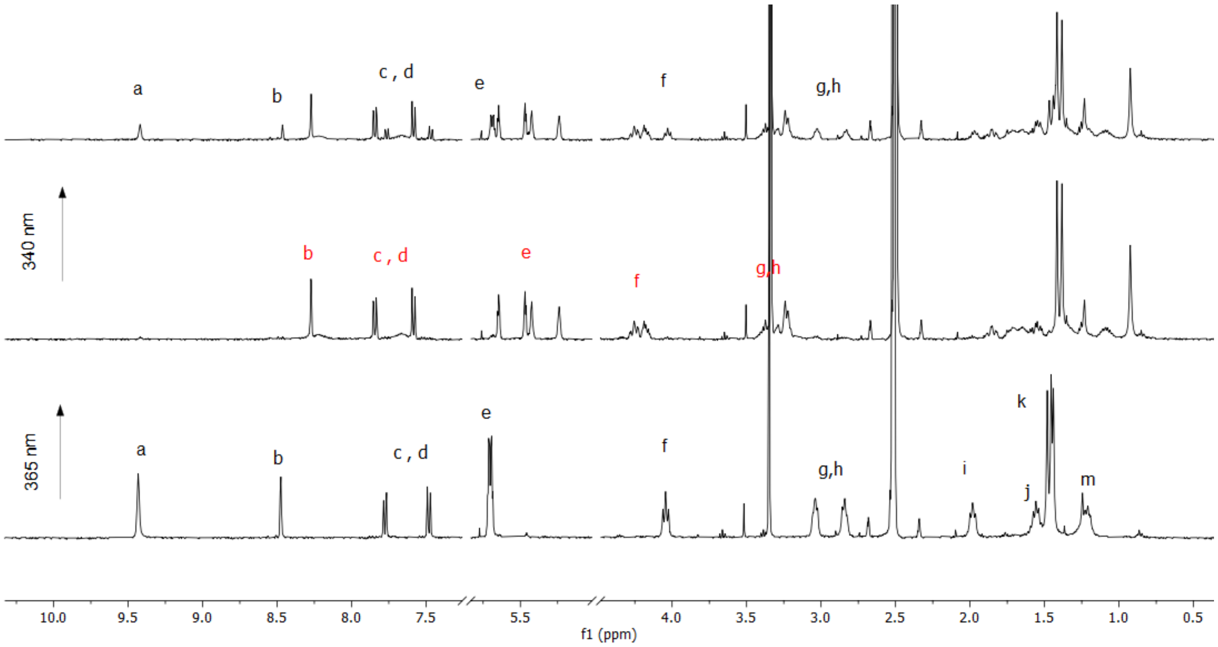

Figure S13. ^{1}H NMR Spectrum (400 MHz, 293 K) of (*Z*)-**1** in degassed DMSO-d_6 (2 mM) (bottom) and spectral changes after irradiation with 365 nm light for 15 min (middle) followed by irradiation with 340 nm light for 2 h (bottom) at 20 °C. For determination of the PSS ratios, the integrals of the H_b, H_c, and H_d signals were averaged giving a PSS_{365} ratio (E/Z) of 99:1 and a PSS_{340} ratio (E/Z) of 73:27.

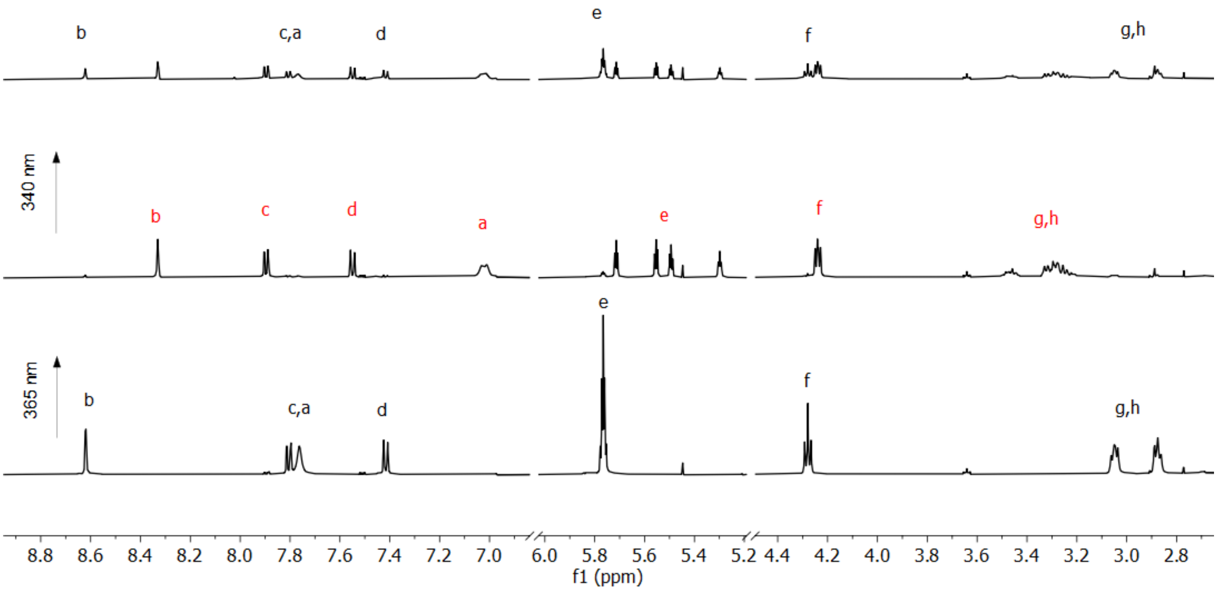

Figure S14. ^{1}H NMR Spectrum (500 MHz, 293 K) of (*Z*)-**1** in degassed MeCN-d_3 (3.8 mM) (bottom) and spectral changes after irradiation with 365 nm light for 15 min (middle) followed by irradiation with 340 nm light for 150 min (bottom) at 20 °C. For determination of the PSS ratios, the integrals of the H_b, H_c, and H_d signals were averaged giving a PSS_{365} ratio (E/Z) of 94:6 and a PSS_{340} ratio (E/Z) of 59:41.

^{1}H NMR thermal stability studies of (*E*)-1

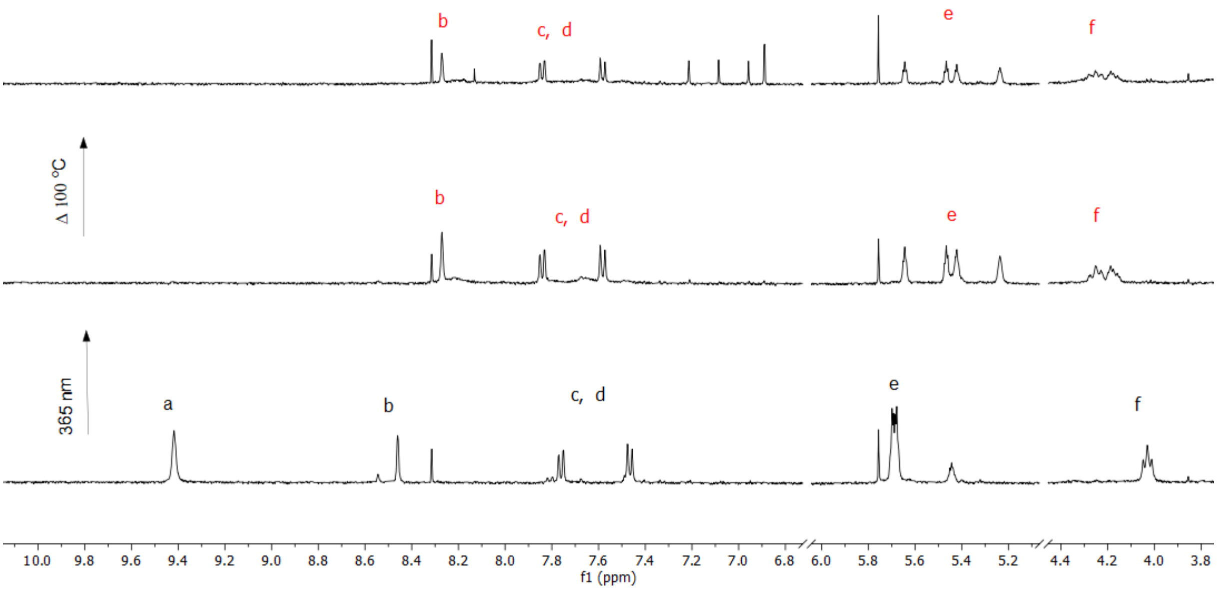

Figure S15. ^{1}H NMR Spectrum (400 MHz, 293 K) of (*Z*)-**1** in degassed DMSO-d_6 (5 mM) (bottom) and spectral changes after irradiation with 365 nm light for 15 min (middle) followed by heating at 100 °C for 5 days in the dark (bottom). The newly appearing signals upon prolonged heating are ascribed to degradation products, which could not be identified.

^{1}H NMR titration experiment of (*Z*)-1

A 2.4 mM solution of receptor (*Z*)-**1** was prepared in DMSO-d_6 and this solution was used to dissolve $[NBu_4]^+[Cl]^-$ at a concentration of 43.2 mM. The chloride solution was added stepwise to the receptor solution to reach the desired number of equivalents and after every addition a ^{1}H NMR spectrum was recorded.

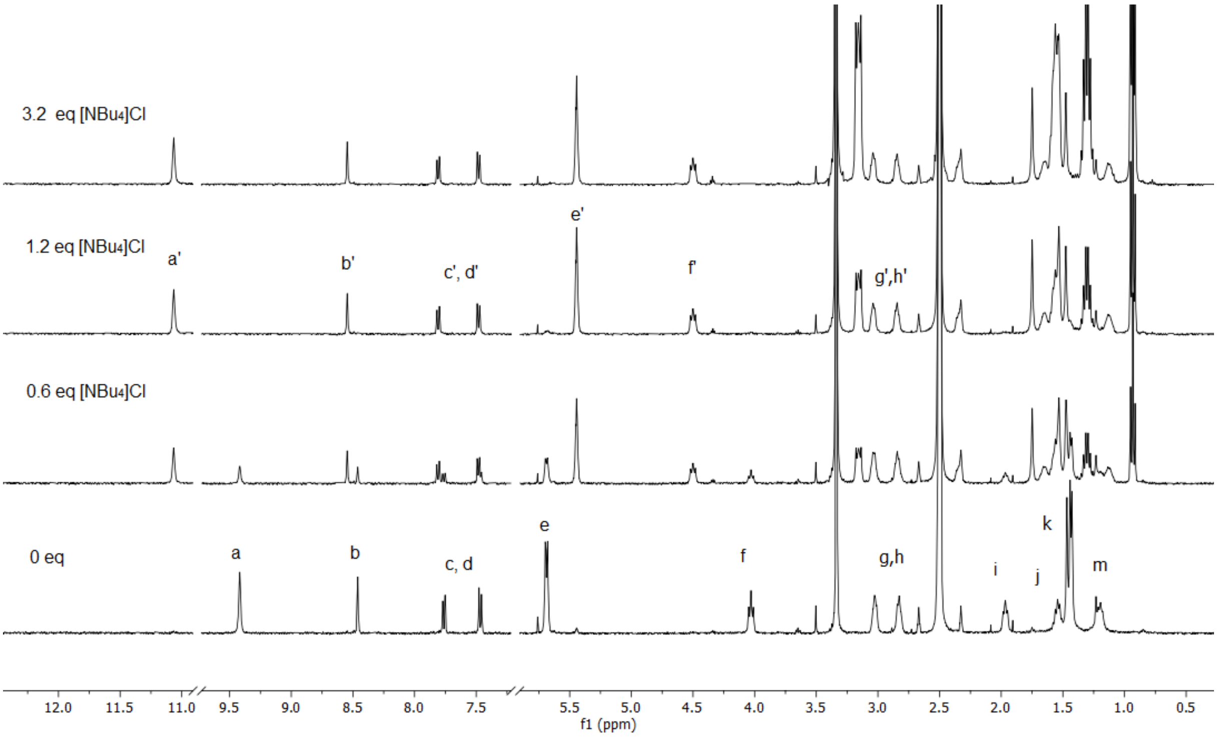

Figure S16. ^{1}H NMR spectral changes (400 MHz, 293 K) of (*Z*)-**1** (2.4 mM in DMSO-d_6) upon the stepwise addition of $[NBu_4]^+[Cl]^-$ (from bottom to top: 0.0, 0.6, 1.2, and 3.2 equiv.).

^{1}H NMR titration experiment of (*E*)-1

First, a 1.7 mM solution of receptor (*Z*)-**1** in degassed DMSO-d_6 was irradiated with 365 nm light until (*E*)-**1** was fully formed (10 min.). The resulting (*E*)-**1** solution was used to prepare two solutions of $[NBu_4]^+[Cl]^-$ at concentrations of 24 mM and 500 mM. The $[NBu_4]^+[Cl]^-$ solutions were added stepwise to the solution of (*E*)-**1** and after every addition a ^{1}H NMR spectrum was recorded.

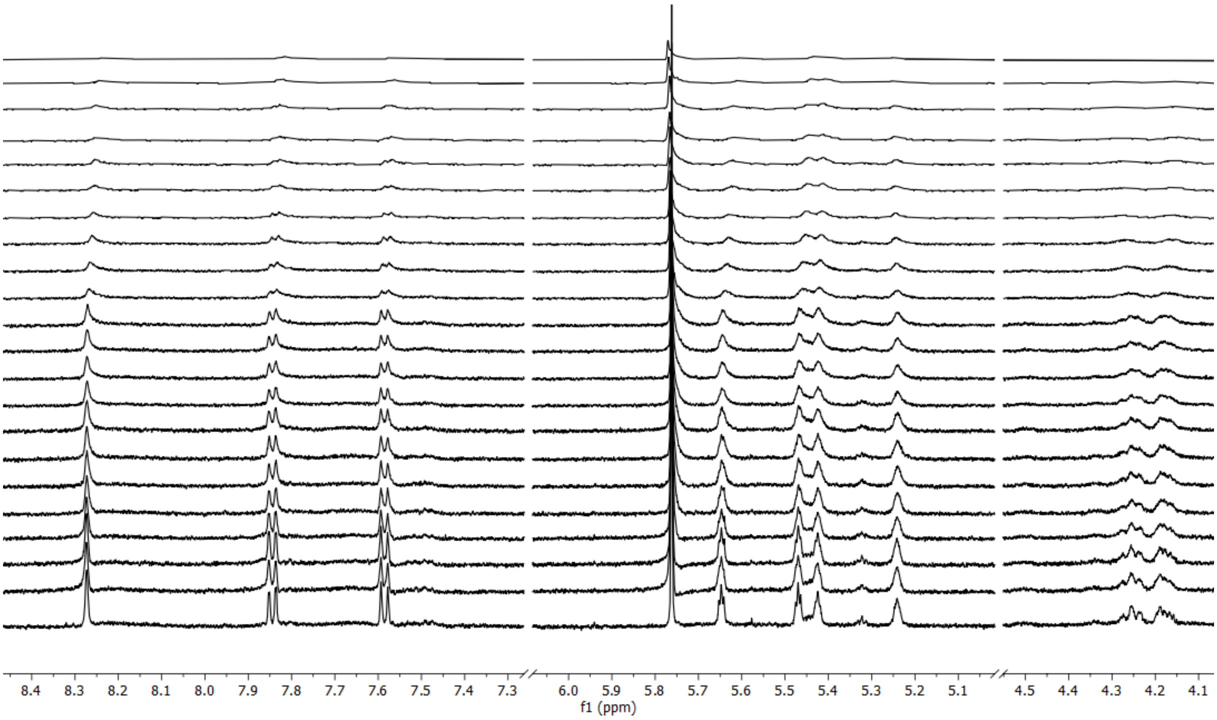

Figure S17. ^{1}H NMR spectral changes (500 MHz, 293 K) of (*E*)-**1** (1.7 mM in DMSO-d_6) upon the stepwise addition of $[NBu_4]^+[Cl]^-$ (from bottom to top: 0.0, 0.3, 0.5, 0.8, 1.1, 1.3, 1.5, 1.7, 1.9, 2.4, 2.7, 7.4, 12.0, 16.4, 20.6, 24.8, 28.8, 32.7, 36.5, 43.72, and 50.6 equiv.).

First, a 1.3 mM solution of receptor (*Z*)-**1** in degassed MeCN-d_3 was irradiated with 365 nm light until (*E*)-**1** was fully formed (15 min.). The resulting (*E*)-**1** solution was used to prepare two solutions of $[NBu_4]^+[Cl]^-$ at concentrations of 24 mM and 500 mM. The $[NBu_4]^+[Cl]^-$ solutions were added stepwise to the solution of (*E*)-**1** and after every addition a ^{1}H NMR spectrum was recorded.

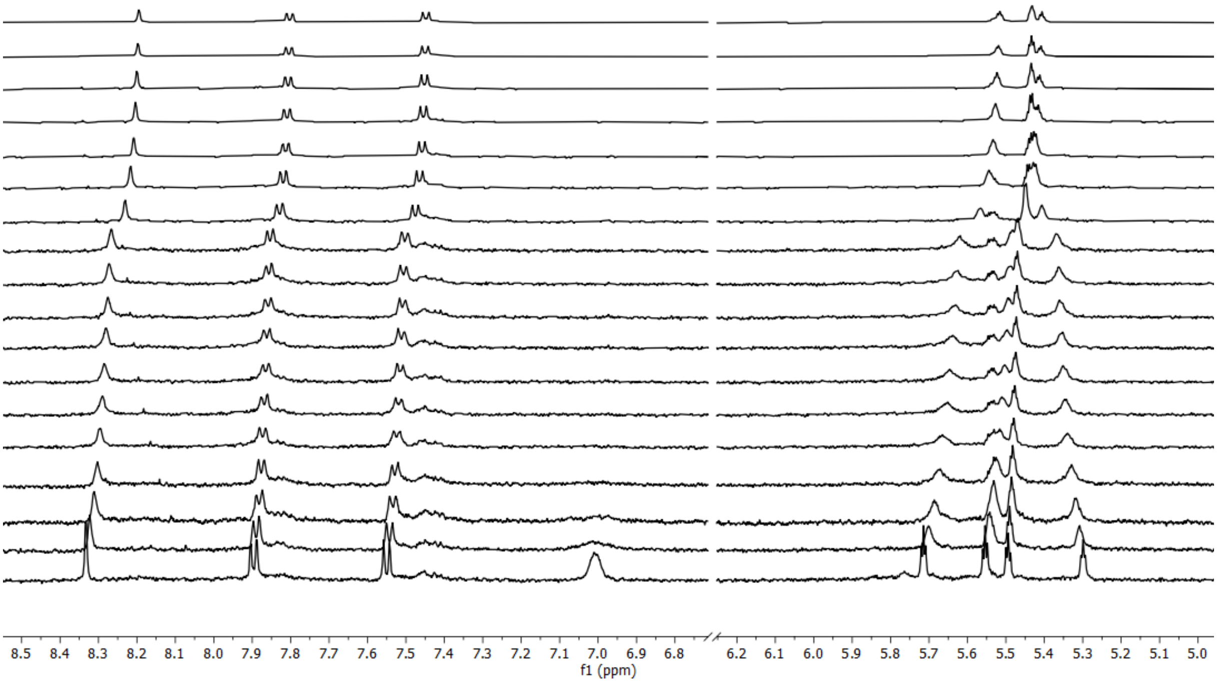

Figure S18. ^{1}H NMR spectral changes (500 MHz, 293 K) of (*E*)-**1** (1.3 mM in MeCN-d_3) upon the stepwise addition of $[NBu_4]^+[Cl]^-$ (from bottom to top: 0.0, 0.4, 0.7, 1.0, 1.4, 1.7, 2.0, 2.3, 2.6, 3.1, 3.6, 9.7, 15.7, 21.5, 27.1, 32.5, 37.8 and 42.9equiv.).

Titration curve analysis

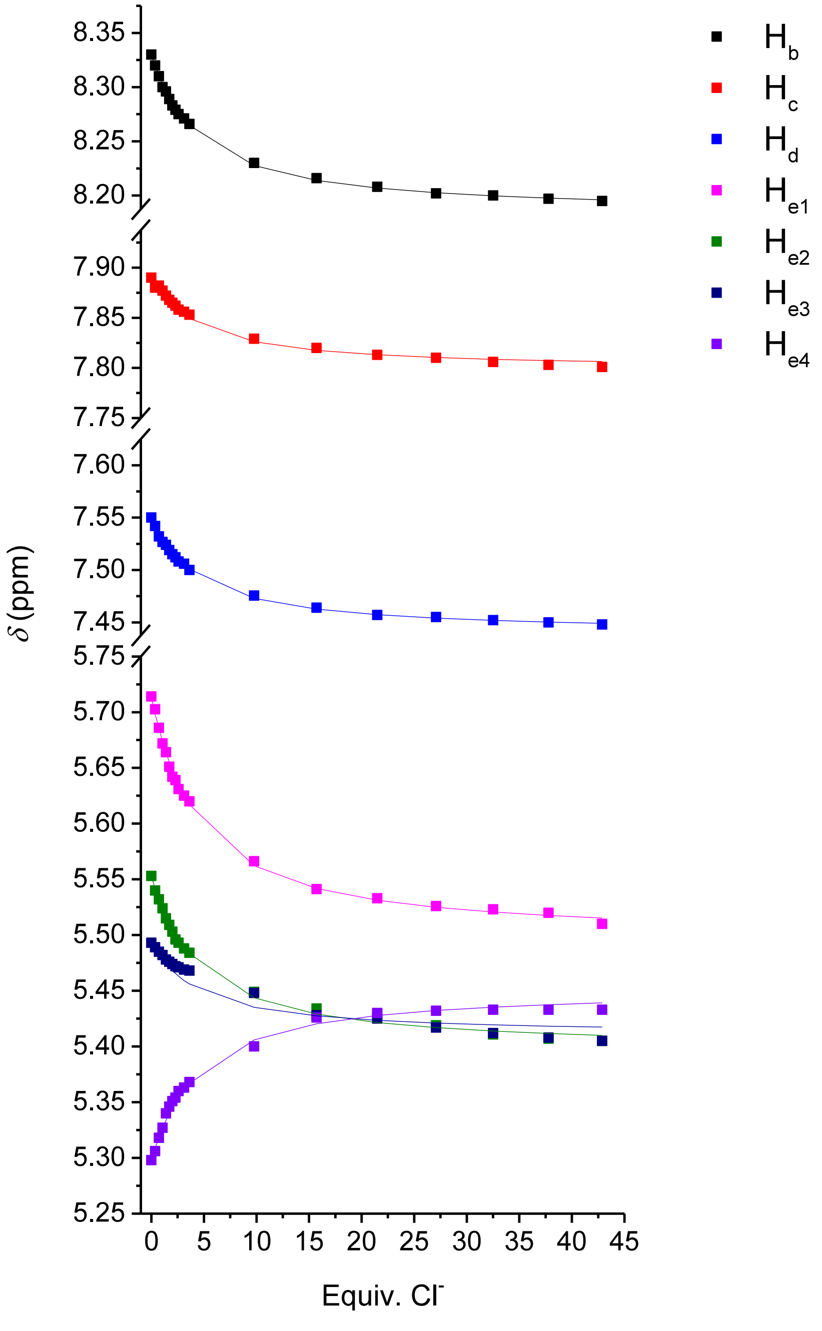

Figure S19. Titration curves for the addition of $[NBu_4]^+[Cl]^-$ to (*E*)-**1** in MeCN-d_3 and data fits obtained using HypNMR[4] by simultaneous analysis of the aromatic (H_b-H_d) and β-pyrrolic signals (H_{e1}-H_{e4}); K_a = 2.0 x 10^2 M^{-1}.

^{1}H NMR photoisomerization of (*Z*)-1⊂Cl$^-$

A 2.4 mM solution of (*Z*)-**1** containing 3.2 equiv. of $[NBu_4]^+[Cl]^-$ in degassed DMSO-d_6 was irradiated with 365 nm light for 15 min, resulting in the formation of non-bound (*E*)-**1** (cf. Figure S13). The non-bound (*E*)-***1*** was further irradiated with 340 nm for 2 h yielding a PSS mixture containing bound (*Z*)-**1** and non-bound (*E*)-**1** species.

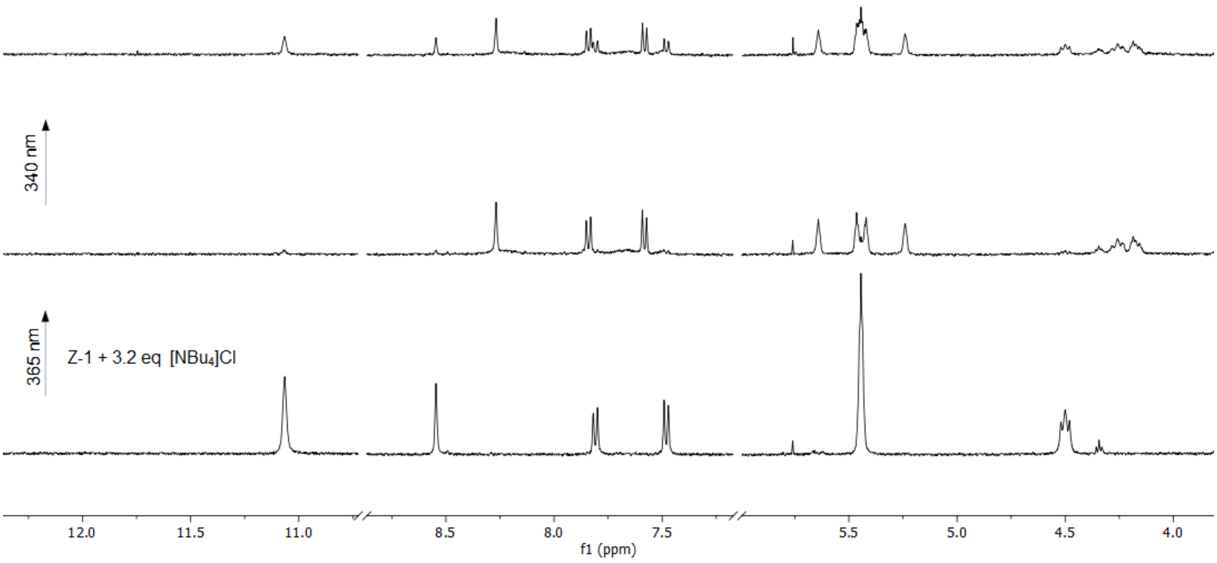

Figure S20. ^{1}H NMR Spectrum (400 MHz, 293 K) of a mixture of (*Z*)-**1** (2.4 mM) and $[NBu_4]^+Cl^-$ (7.68 mM) in degassed DMSO-d_6 (bottom) and spectral changes after irradiation with 365 nm light for 15 min (middle) followed by irradiation with 340 nm light for 2 h (bottom) at 20 °C. The middle spectrum is virtually the same as that of (*E*)-**1** with post-added $[NBu_4]^+[Cl]^-$ (see Figure S17). For determination of the PSS ratios, the integrals of the H_b, H_c, and H_d signals were averaged giving a PSS_{365} ratio (*E*/*Z*) of 99:1 and a PSS_{340} ratio (*E*/*Z*) of 67:33, slightly higher that the PSS in absence of $[NBu_4]^+$ $[Cl^-]$, i.e. PSS_{340} = 73:27 (*E*/*Z*).

NMR dilution experiment of (*E*)-1

A 1.68 mM solution of (*Z*)-**1** in degassed DMSO-d_6 was irradiated with 365 nm light until (*E*)-**1** was fully formed (10 min). Subsequently, 0.5 mL of DMSO-d_6 was added three times and after every dilution a ^{1}HNMR spectrum was recorded.

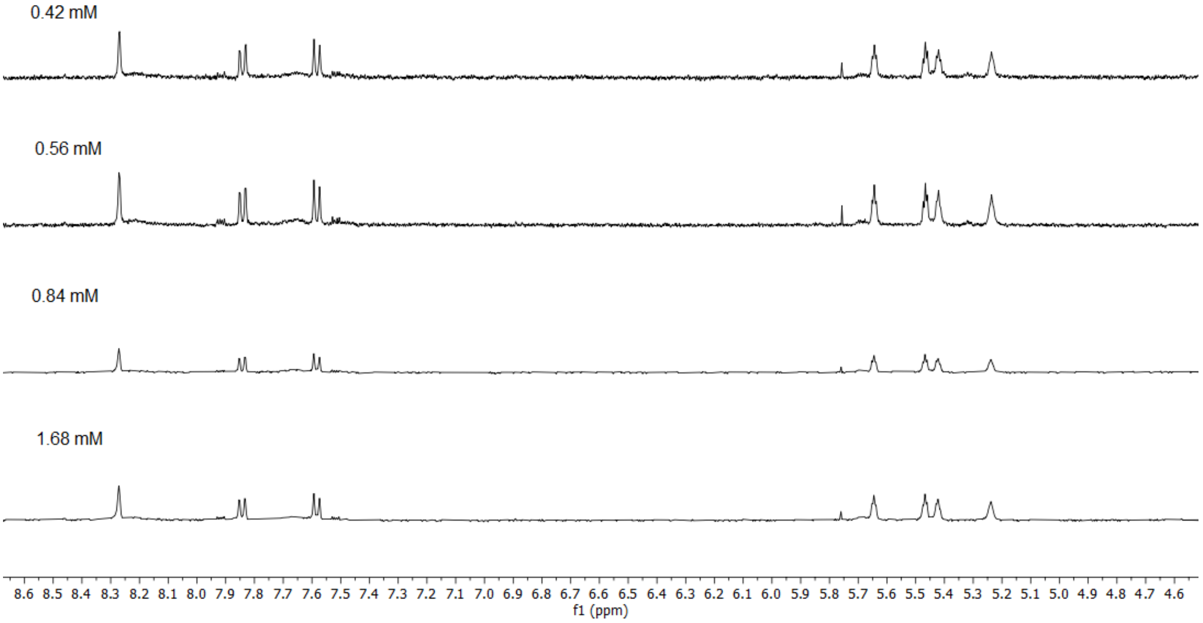

Figure S21. ^{1}H NMR spectra (400 MHz, 293 K) of (*E*)-**1** at various concentrations, starting from 1.68 mM (bottom) to 0.42 mM (top).

ITC titration experiments

The titrations were carried out in dry acetonitrile at 25 °C. For the titration with (*Z*)-**1**, a 1.20 mM solution was prepared. For (*E*)-**1**, a 1.26 mM solution of (*Z*)-**1** was prepared and the sample was irradiated in a 1 mm quartz cuvette until UV-Vis spectroscopy confirmed full conversion (8 min). Aliquots of 6 μL of a solution of either (*Z*)-**1** or (*E*)-**1**) were titrated into a 0.06 mM solution of $[NBu_4]^+[Cl]^-$. To calculate the association constant and thermodynamic parameters, the data was fitted to a 1:1 binding model using Origin software.

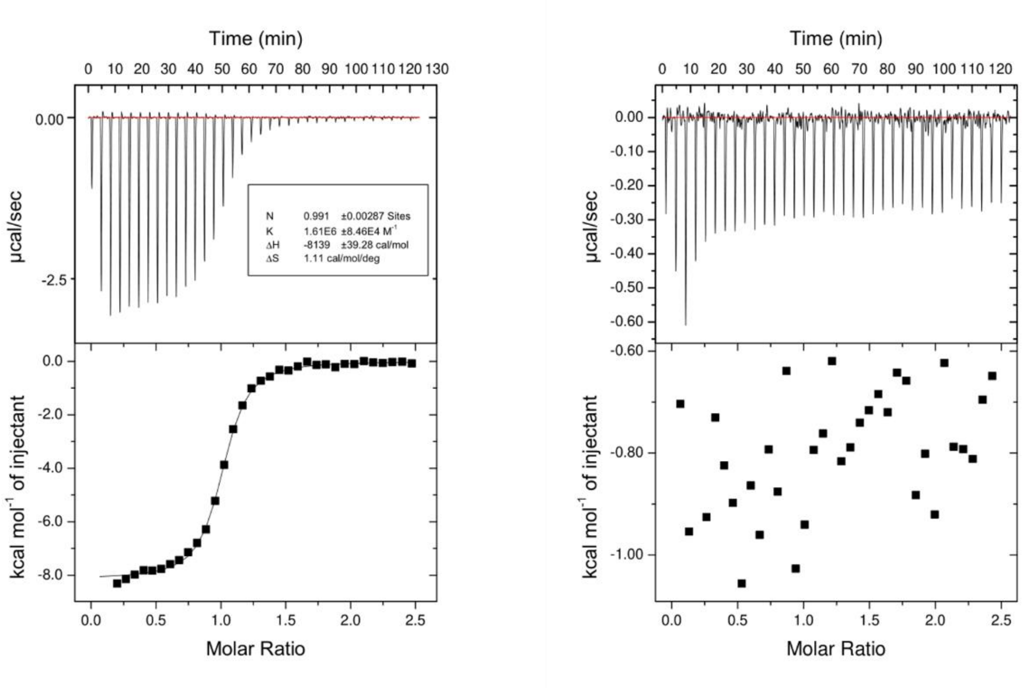

Figure S22. Isothermal calorimetric titration in acetonitrile at 298 K of host (*Z*)-**1** at 1.20 mM (left) or (*E*)-**1** at 1.26 mM (right) titrated into $[NBu_4]^+[Cl]^-$ solution at 0.06 mM.

Single crystal X-ray crystallography

All reflection intensities were measured at 110(2) K using a SuperNova diffractometer (equipped with Atlas detector) with Cu *K*α radiation (λ = 1.54178 Å) under the program CrysAlisPro (Version CrysAlisPro 1.171.39.29c, Rigaku OD, 2017). The same program was used to refine the cell dimensions and for data reduction. The structure was solved with the program SHELXS-2018/3 (Sheldrick, 2018) and was refined on F^2 with SHELXL-2018/3 (Sheldrick, 2018). Analytical numeric absorption correction based using a multifaceted crystal was applied using CrysAlisPro. The temperature of the data collection was controlled using the Cryojet system (manufactured by Oxford Instruments). The H atoms were placed at calculated positions using the instructions AFIX 23, AFIX 43 or AFIX 137 with isotropic displacement parameters having values 1.2 or 1.5 U_{eq} of the attached C atoms.

The structure is mostly ordered. The atom O3 is disordered over two positions, and the occupancy factor of the major component of the disorder refines to 0.635(4).

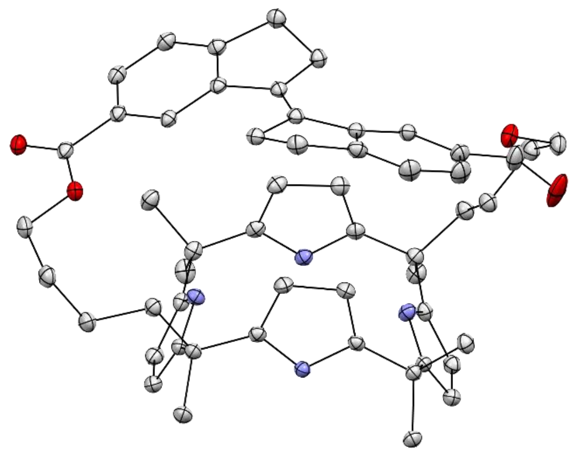

Figure S23. Displacement ellipsoid shown at the 50% probability level of (*E*)-**1**.

Table S1. Crystallographic data for the structure of (*E*)-1.

	(*E*)-**1**
Crystal data	
Chemical formula	$C_{54}H_{60}N_4O_4{\cdot}C_2H_3N$
M_r	870.11
Crystal system, space group	Monoclinic, $P2_1/c$
Temperature (K)	110
a, *b*, *c* (Å)	14.7734 (2), 17.4936 (3), 18.6427 (3)
β (°)	90.2338 (13)
V (Å^3)	4817.98 (13)
Z	4
Radiation type	Cu *K*α
μ (mm^{-1})	0.59
Crystal size (mm)	0.25 × 0.16 × 0.13
Data collection	
Diffractometer	SuperNova, Dual, Cu at zero, Atlas
Absorption correction	Analytical<br>*CrysAlis PRO* 1.171.39.46 (Rigaku Oxford Diffraction, 2018) Analytical numeric absorption correction using a multifaceted crystal model based on expressions derived by R.C. Clark & J.S. Reid. (Clark, R. C. & Reid, J. S. (1995). Acta Cryst. A51, 887-897) Empirical absorption correction using spherical harmonics, implemented in SCALE3 ABSPACK scaling algorithm.
T_{min}, T_{max}	0.895, 0.945
No. of measured, independent and observed [$I > 2\sigma(I)$] reflections	37786, 9426, 8076
R_{int}	0.026
$(\sin \theta/\lambda)_{max}$ (Å^{-1})	0.616
Refinement	
$R[F^2 > 2\sigma(F^2)]$, $wR(F^2)$, S	0.041, 0.109, 1.04
No. of reflections	9426
No. of parameters	609
No. of restraints	5
H-atom treatment	H atoms treated by a mixture of independent and constrained refinement
$\Delta\rho_{max}$, $\Delta\rho_{min}$ (e Å^{-3})	0.32, -0.32

Computer programs: *CrysAlis PRO* 1.171.39.29c (Rigaku OD, 2017), *SHELXS2018*/3 (Sheldrick, 2018), *SHELXL2018*/3 (Sheldrick, 2018), *SHELXTL* v6.10 (Sheldrick, 2008).[5]

Geometry optimization by DFT

Input geometries were generated using ArgusLab.[6] For the energy minimization of (*M*)-(*E*)-**1**⊂Cl⁻, the single crystal X-ray structure of (*M*)-(*E*)-**1** was modified and used as the starting geometry. For (*P*)-(*Z*)-**1**⊂Cl⁻, the calix[4]pyrrole-alkyl and stiff-stilbene-CO_2Me fragments were generated and energy minimized first, after which the latter fragment was positioned on top of the first fragment. The Gaussian 09 program[7] was used for further geometry optimization. Initially, energy minimizations were performed at the semi-empirical PM3 and the DFT B3LYP/STO3G levels of theory using tight convergence criteria. Subsequently, the lowest energy geometries were optimized at the DFT B3LYP/6-31G+(d,p) level of theory using an IEFPCM MeCN solvation model. The DFT-optimized geometry was found to have <u>zero imaginary frequencies</u>.

Table S2. Cartesian coordinates of (*P*)-(*Z*)-**1**⊂Cl⁻.

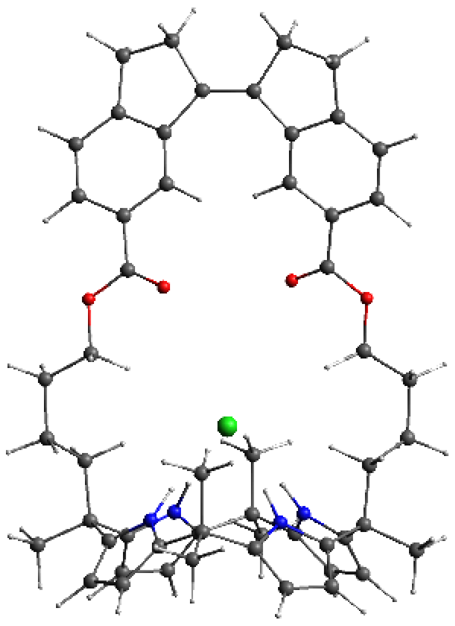

atom	X	Y	Z
Cl	1.1130900000	-0.0000300000	-0.0000400000
N	3.4762300000	1.9940800000	-1.3724500000
N	3.4881700000	-1.3810700000	-1.9751300000
N	3.4762400000	-1.9940500000	1.3724900000
N	3.4880200000	1.3811000000	1.9751700000
C	4.0612200000	3.0724900000	-0.7341500000

C	5.0607800000	3.5358400000	-1.5744000000
C	5.0702800000	2.7136900000	-2.7406900000
C	4.0757100000	1.7599100000	-2.5972900000
C	3.6360600000	0.6396500000	-3.5372200000
C	4.0946000000	-0.7269600000	-3.0320500000
C	5.1108400000	-1.5554100000	-3.4798600000
C	5.1067100000	-2.7310900000	-2.6713100000
C	4.0882600000	-2.6055200000	-1.7395300000
C	3.6232200000	-3.5662500000	-0.6428500000
C	4.0613000000	-3.0724500000	0.7342200000
C	5.0608400000	-3.5357500000	1.5745200000
C	5.0702500000	-2.7136000000	2.7408100000
C	4.0756500000	-1.7598600000	2.5973500000
C	3.6359100000	-0.6396100000	3.5372700000
C	4.0944300000	0.7270100000	3.0321200000
C	5.1106200000	1.5554900000	3.4799800000
C	5.1064800000	2.7311800000	2.6714300000
C	4.0880900000	2.6055700000	1.7396000000
C	3.6230600000	3.5662900000	0.6429000000
C	2.0675200000	-3.7114000000	-0.7299000000
C	4.2757510000	4.9413000000	0.9030000000
C	4.2616900000	0.8866000000	-4.9266300000
C	2.0930300000	0.6596600000	-3.7089100000
C	4.2759600000	-4.9412500000	-0.9029300000
C	4.2614900000	-0.8865500000	4.9267100000
C	2.0928800000	-0.6596900000	3.7088900000
C	2.0673500000	3.7113900000	0.7298800000
C	1.4147010000	4.6992800000	-0.2515100000
C	1.4148400000	-4.6992800000	0.2514800000
C	-0.1199090000	4.7560600000	-0.1074500000
C	-0.1197700000	-4.7560500000	0.1073800000
C	-0.8214200000	-3.5519800000	0.7208800000
C	-0.8215600000	3.5520200000	-0.7210100000
O	-2.6140800000	1.6620800000	-1.3894200000
O	-2.6139500000	-1.6620400000	1.3892600000
C	-6.1997500000	3.9700700000	0.8619400000
C	-4.8765300000	3.8927000000	0.4214700000
C	-4.4051300000	2.7159800000	-0.1874900000
C	-7.0362400000	2.8632300000	0.7230400000
C	-6.5585400000	1.6565300000	0.1633200000
C	-5.2481800000	1.6031300000	-0.3240400000
C	-5.2480900000	-1.6031600000	0.3239400000
C	-4.4049900000	-2.7159700000	0.1873400000
C	-4.8763700000	-3.8927200000	-0.4215800000
C	-6.5584800000	-1.6566200000	-0.1633400000
C	-7.0361500000	-2.8633500000	-0.7230300000
C	-6.1996200000	-3.9701500000	-0.8619800000
C	-7.6592500000	-0.6711200000	-0.0989800000
C	-8.4948700000	-2.7382500000	-1.0895200000
C	-8.9456900000	-1.4657100000	-0.3290300000

C	-8.4949300000	2.7380700000	1.0896200000
C	-7.6592800000	0.6709800000	0.0990300000
C	-8.9457400000	1.4655000000	0.3291600000
C	-3.0123900000	-2.5978300000	0.7078800000
C	-3.0125500000	2.5979100000	-0.7081200000
O	-2.2245200000	-3.6227500000	0.3373700000
O	-2.2246800000	3.6227900000	-0.3375500000
H	5.7123710000	4.3768100000	-1.3826300000
H	5.7299900000	2.8207900000	-3.5903200000
H	5.7801800000	-1.3521800000	-4.3040500000
H	5.7731100000	-3.5755800000	-2.7760200000
H	5.7124700000	-4.3767000000	1.3827700000
H	5.7299300000	-2.8206800000	3.5904700000
H	5.7799300000	1.3523000000	4.3042000000
H	5.7728400000	3.5756900000	2.7761700000
H	1.8301200000	-4.0247800000	-1.7551800000
H	1.6076900000	-2.7264500000	-0.6007900000
H	3.9364110000	5.3431000000	1.8623500000
H	5.3656610000	4.8688200000	0.9319600000
H	4.0136610000	5.6525500000	0.1171000000
H	5.3542900000	0.8888500000	-4.8912800000
H	3.9462400000	0.1048800000	-5.6234700000
H	3.9329000000	1.8530600000	-5.3192500000
H	1.5615400000	0.4979400000	-2.7676300000
H	1.7769300000	1.6281100000	-4.1110300000
H	1.7847400000	-0.1238800000	-4.4092900000
H	5.3658700000	-4.8687300000	-0.9318300000
H	4.0138600000	-5.6525000000	-0.1170300000
H	3.9366800000	-5.3430600000	-1.8622900000
H	5.3541000000	-0.8887500000	4.8914000000
H	3.9459800000	-0.1048300000	5.6235300000
H	3.9327100000	-1.8530200000	5.3193100000
H	1.5614300000	-0.4979900000	2.7675800000
H	1.7768000000	-1.6281500000	4.1109900000
H	1.7845200000	0.1238500000	4.4092500000
H	2.7063300000	1.4454500000	-0.9931300000
H	2.7083100000	-1.0056400000	-1.4385700000
H	2.7063400000	-1.4454500000	0.9931300000
H	2.7082000000	1.0056300000	1.4385700000
H	1.8299000000	4.0247500000	1.7551500000
H	1.6075600000	2.7264400000	0.6007300000
H	1.8039510000	5.7075000000	-0.0772400000
H	1.6822310000	4.4399600000	-1.2842500000
H	1.6823400000	-4.4399500000	1.2842200000
H	1.8040900000	-5.7075000000	0.0772200000
H	-0.3847990000	4.8180600000	0.9558000000
H	-0.5021690000	5.6666700000	-0.5835000000
H	-0.5020500000	-5.6666500000	0.5834400000
H	-0.3846300000	-4.8180800000	-0.9558800000
H	-0.4184300000	2.6012600000	-0.3637300000

H	-0.7575300000	3.5624600000	-1.8136300000
H	-0.7574200000	-3.5623900000	1.8135000000
H	-0.4182600000	-2.6012400000	0.3635700000
H	-6.5733390000	4.8917000000	1.2996300000
H	-4.8635300000	0.7254900000	-0.8267300000
H	-4.8634500000	-0.7255100000	0.8266200000
H	-6.5732000000	-4.8917900000	-1.2996500000
H	-8.6013400000	-2.5961600000	-2.1735800000
H	-9.6991600000	-0.8946500000	-0.8771500000
H	-9.3913300000	-1.7494900000	0.6351300000
H	-9.0739100000	3.6278000000	0.8245900000
H	-8.6013300000	2.5959700000	2.1736900000
H	-9.3914400000	1.7492700000	-0.6349700000
H	-9.6991500000	0.8944100000	0.8773300000
H	-4.2160390000	4.7455600000	0.5263100000
H	-4.2158600000	-4.7455400000	-0.5264700000
H	-9.0738000000	-3.6280000000	-0.8244500000

Sum of electronic and zero-point Energies = -3073.373589

Table S3. Cartesian coordinates of (*M*)-(*E*)-**1**⊂Cl^-.

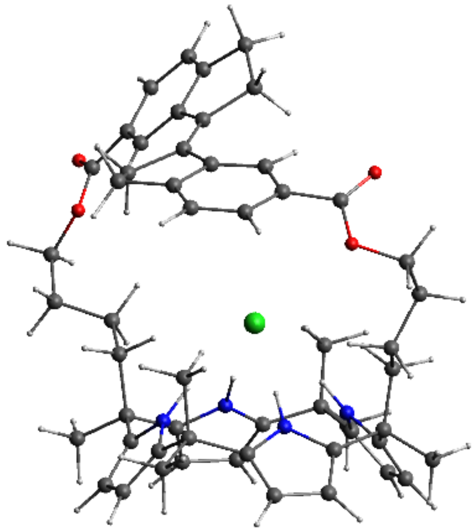

atom	X	Y	Z
O	-5.3348420000	-2.3668650000	0.5520600000
O	1.8962650000	5.3717980000	0.0196500000
N	3.6870600000	0.2745270000	0.7737200000
N	1.4597680000	-1.9141110000	2.2128600000
O	0.2742660000	5.7924700000	-1.5061800000
N	2.9486690000	-0.9853430000	-2.2802200000
N	0.6958070000	-3.1588210000	-0.8446500000
C	2.4641190000	-1.6109520000	3.1139800000

C	-2.3254060000	3.5700820000	1.0506000000
C	1.5131370000	-2.8695210000	-3.2529300000
C	4.6858810000	0.7527660000	-1.5407100000
C	0.6140150000	5.4092500000	-0.3944600000
C	-1.5034060000	4.2630820000	0.1548800000
C	4.2472100000	-0.5054740000	-2.2942600000
C	-4.2299970000	3.5596240000	-1.5051100000
C	4.0799300000	0.0347260000	2.0795900000
C	-0.0628140000	-3.8463400000	1.5023200000
C	-3.4610070000	2.6671030000	0.7904100000
C	6.0860410000	1.1521940000	-2.0587600000
C	-0.8556760000	4.1908910000	2.8978900000
C	2.8333280000	-2.1261630000	-3.0541200000
C	3.0947200000	-0.2233930000	3.2177200000
C	1.1504670000	-3.7093410000	-2.0298100000
C	5.9055300000	0.3703040000	0.7675400000
C	-6.0078580000	1.9569860000	-1.7430000000
C	2.9287450000	5.6414870000	-0.9734800000
C	5.4639400000	0.0889950000	2.0941400000
C	-6.2550210000	-0.6398540000	-0.7441800000
C	1.1818550000	-5.0818110000	-1.8437100000
C	-0.3260150000	4.8650000000	0.6248700000
C	4.7857010000	0.4825160000	-0.0408800000
C	1.6561660000	-3.8085810000	-4.4705600000
C	-5.3242400000	0.2379650000	-0.1658400000
C	-4.2147470000	2.5816340000	-0.3336200000
C	3.6904020000	1.9154170000	-1.8713400000
C	-1.9878470000	3.5328820000	2.4219200000
C	4.9767790000	-1.3684850000	-3.0970700000
C	-5.2040880000	1.5403250000	-0.6599600000
C	-7.0905200000	-0.1977030000	-1.7842200000
C	1.0513670000	-3.2295210000	2.3491700000
C	-6.2919120000	-2.0766340000	-0.3444300000
C	-2.9676270000	2.6667330000	3.1763000000
C	0.4344160000	-4.1453400000	0.0897000000
C	0.7326550000	-5.3546110000	-0.5171800000
C	-5.6210070000	3.3544750000	-2.1673500000
C	-2.5412030000	-3.3172470000	0.7116600000
C	-0.0078250000	4.8369100000	1.9935000000
C	1.9913310000	0.8683780000	3.2315500000
C	1.8217260000	-3.7787120000	3.3616300000
C	-0.5018750000	-5.1694590000	2.1636800000
C	4.0926280000	-2.3822240000	-3.5718800000
C	3.8598730000	3.2310960000	-1.0893600000
C	3.1913340000	4.4045570000	-1.8299100000
C	-3.6275280000	1.8249640000	2.0533300000
C	-5.0599340000	-3.7528650000	0.8889000000
C	-3.7155840000	-3.7624060000	1.6067300000
C	-1.2764230000	-2.8597290000	1.4789000000
C	0.3724680000	-1.8660500000	-3.5727500000

C	2.7061870000	-2.7659620000	3.8400100000
C	-6.9676890000	1.1024570000	-2.2849200000
O	-7.0652730000	-2.9099130000	-0.7999800000
C	3.8553100000	-0.1287340000	4.5583200000
Cl	0.4866700000	0.1726600000	-0.2823300000
H	-1.7038460000	4.2747220000	-0.9091800000
H	-3.4442370000	3.3025130000	-2.2290600000
H	-4.0605460000	4.5938640000	-1.1937600000
H	6.8201600000	0.3599540000	-1.8927500000
H	6.4474620000	2.0501740000	-1.5527200000
H	6.0423810000	1.3546340000	-3.1325300000
H	-0.6033460000	4.1639710000	3.9542900000
H	6.9324810000	0.4803030000	0.4495400000
H	3.8020160000	5.9219960000	-0.3805000000
H	2.6158360000	6.4905880000	-1.5842100000
H	6.0959200000	-0.0501760000	2.9598200000
H	1.4840950000	-5.8156310000	-2.5775300000
H	0.7167960000	-4.3410510000	-4.6448900000
H	2.4453560000	-4.5517920000	-4.3292600000
H	1.8947170000	-3.2267120000	-5.3653300000
H	-4.6727400000	-0.1271150000	0.6149700000
H	2.6582820000	1.5781080000	-1.7276700000
H	3.7952920000	2.1131760000	-2.9466300000
H	6.0278590000	-1.2817360000	-3.3330500000
H	-7.8036110000	-0.8898420000	-2.2194000000
H	-2.4883580000	2.0548220000	3.9458100000
H	0.6360840000	-6.3319710000	-0.0656300000
H	-5.5993670000	3.4758650000	-3.2543500000
H	-6.3438260000	4.0830360000	-1.7755800000
H	-2.2907040000	-4.1216380000	0.0084400000
H	-2.8871920000	-2.4791170000	0.0979600000
H	0.9091450000	5.2991490000	2.3416400000
H	2.4481620000	1.8559780000	3.3543700000
H	1.3068310000	0.6967690000	4.0691300000
H	1.4010210000	0.8857290000	2.3119100000
H	1.7549850000	-4.7918420000	3.7322900000
H	-1.2889050000	-5.6504490000	1.5778800000
H	-0.8857450000	-4.9767590000	3.1695200000
H	0.3279140000	-5.8762000000	2.2464500000
H	4.3548170000	-3.1983340000	-4.2304200000
H	3.4174730000	3.1192770000	-0.0930700000
H	4.9189730000	3.4679950000	-0.9342200000
H	2.2388140000	4.0827180000	-2.2674500000
H	3.8235750000	4.7128460000	-2.6731100000
H	-4.6729080000	1.5839850000	2.2638800000
H	-5.0453140000	-4.3391950000	-0.0348800000
H	-5.8665440000	-4.1250340000	1.5276800000
H	-3.7921330000	-3.0990660000	2.4776700000
H	-3.5514140000	-4.7729460000	1.9991900000
H	-1.5351920000	-2.6457880000	2.5236200000

H	-0.9448220000	-1.9074790000	1.0552800000
H	0.6225390000	-1.2903700000	-4.4703600000
H	0.1903190000	-1.1587300000	-2.7596100000
H	-0.5594120000	-2.4105490000	-3.7587500000
H	3.4263670000	-2.8755630000	4.6385400000
H	-7.5933890000	1.4262970000	-3.1120500000
H	4.6580990000	-0.8673640000	4.6302100000
H	3.1664700000	-0.2941830000	5.3915000000
H	4.2981910000	0.8652660000	4.6684000000
H	2.7183500000	0.3147880000	0.4600200000
H	1.0798390000	-1.2564610000	1.5352000000
H	2.1803800000	-0.5645120000	-1.7606500000
H	0.5629480000	-2.1612000000	-0.6874200000
H	-3.7179370000	3.2919540000	3.6794700000
H	-3.0844090000	0.8753030000	1.9540600000

Sum of electronic and zero-point Energies = -3073.368837

References

(1) D. C. Horwell, W. Howson, W. P. Nolan, G. S. Ratcliffe, D. C. Rees, H. M. G. Willems, *Tetrahedron*, 1995, **51**, 203-206.

(2) A. K. Ghosh, D. R. Nicponski, *Org. Lett.*, 2011, **13**, 4328-4331.

(3) S. Akbulatov, T. Yancong, R. Boulatov, *J. Am. Chem. Soc.*, 2012, **134**, 7620-7623.

(4) C. Frassineti, S. Ghelli, P. Gans, A. Sabatini, M. S. Moruzzi, A. Vacca, *Anal. Biochem.*, 1995, **231**, 374-382.

(5) G. M. Sheldrick, *Acta Cryst.*, 2015, **C71**, 3-8.

(6) http://www.arguslab.com/arguslab.com/ArgusLab.html

(7) Gaussian 09, Revision D.01, M. J. Frisch, G. W. Trucks, H. B. Schlegel, G. E. Scuseria, M. A. Robb, J. R. Cheeseman, G. Scalmani, V. Barone, B. Mennucci, G. A. Petersson, H. Nakatsuji, M. Caricato, X. Li, H. P. Hratchian, A. F. Izmaylov, J. Bloino, G. Zheng, J. L. Sonnenberg, M. Hada, M. Ehara, K. Toyota, R. Fukuda, J. Hasegawa, M. Ishida, T. Nakajima, Y. Honda, O. Kitao, H. Nakai, T. Vreven, J. A. Montgomery, Jr., J. E. Peralta, F. Ogliaro, M. Bearpark, J. J. Heyd, E. Brothers, K. N. Kudin, V. N. Staroverov, T. Keith, R. Kobayashi, J. Normand, K. Raghavachari, A. Rendell, J. C. Burant, S. S. Iyengar, J. Tomasi, M. Cossi, N. Rega, J. M. Millam, M. Klene, J. E. Knox, J. B. Cross, V. Bakken, C. Adamo, J. Jaramillo, R. Gomperts, R. E. Stratmann, O. Yazyev, A. J. Austin, R. Cammi, C. Pomelli, J. W. Ochterski, R. L. Martin, K. Morokuma, V. G. Zakrzewski, G. A. Voth, P. Salvador, J. J. Dannenberg, S. Dapprich, A. D. Daniels, O. Farkas, J. B. Foresman, J. V. Ortiz, J. Cioslowski,, D. J. Fox, Gaussian, Inc., Wallingford CT, **2013**.
